# Pathway-Based Evaluation in Early Onset Colorectal Cancer Suggests Focal Adhesion and Immunosuppression along with Epithelial-Mesenchymal Transition

**DOI:** 10.1371/journal.pone.0031685

**Published:** 2012-04-09

**Authors:** Seungyoon Nam, Taesung Park

**Affiliations:** 1 Cancer Genomics Branch, Research Institute, National Cancer Center, Goyang, Korea; 2 Supercomputing Center, Korea Institute of Science and Technology Information, Daejeon, Korea; 3 Department of Statistics, Seoul National University, Seoul, Korea; 4 Interdisciplinary Program in Bioinformatics, Seoul National University, Seoul, Korea; Michigan State University, United States of America

## Abstract

Colorectal cancer (CRC) has one of the highest incidences among all cancers. The majority of CRCs are sporadic cancers that occur in individuals without family histories of CRC or inherited mutations. Unfortunately, whole-genome expression studies of sporadic CRCs are limited. A recent study used microarray techniques to identify a predictor gene set indicative of susceptibility to early-onset CRC. However, the molecular mechanisms of the predictor gene set were not fully investigated in the previous study. To understand the functional roles of the predictor gene set, in the present study we applied a subpathway-based statistical model to the microarray data from the previous study and identified mechanisms that are reasonably associated with the predictor gene set. Interestingly, significant subpathways belonging to 2 KEGG pathways (focal adhesion; natural killer cell-mediated cytotoxicity) were found to be involved in the early-onset CRC patients. We also showed that the 2 pathways were functionally involved in the predictor gene set using a text-mining technique. Entry of a single member of the predictor gene set triggered a focal adhesion pathway, which confers anti-apoptosis in the early-onset CRC patients. Furthermore, intensive inspection of the predictor gene set in terms of the 2 pathways suggested that some entries of the predictor gene set were implicated in immunosuppression along with epithelial-mesenchymal transition (EMT) in the early-onset CRC patients. In addition, we compared our subpathway-based statistical model with a gene set-based statistical model, MIT Gene Set Enrichment Analysis (GSEA). Our method showed better performance than GSEA in the sense that our method was more consistent with a well-known cancer-related pathway set. Thus, the biological suggestion generated by our subpathway-based approach seems quite reasonable and warrants a further experimental study on early-onset CRC in terms of dedifferentiation or differentiation, which is underscored in EMT and immunosuppression.

## Introduction

Familial adenomatous polyposis (FAP) and hereditary nonpolyposis colorectal cancer (HNPCC) are autosomal dominant diseases that result from inherited genetic mutations in adenomatous polyposis coli (APC) and mismatch repair genes [1]. However, these diseases account for only 25% of the total number of colorectal cases in the United States in 2010 [2]. The remaining 75% of cancers are reportedly sporadic colorectal cancers (CRCs) without family histories [2] (www.cancer.gov), for which the mechanism is still not clear [3].

Hong et al. [3] identified 7 highly upregulated genes (*CYR61*, *EGR1*, *FOSB*, *FOS*, *VIP*, *UCHL1*, *KRT24*) in early onset sporadic CRC patients that were used as a predictor gene set assessed with a microarray technique. For their experiments, normal-appearing mucosa adjacent to tumor was obtained from the CRC patients and normal mucosa was obtained from healthy controls. They also provided a discussion on signaling pathways (MAP kinase (MAPK) signaling, NFAT-immune signaling, hypoxia signaling, insulin signaling, PI3K-AKT signaling, Wnt signaling, G protein-coupled receptor (GPCR) signaling).

In the present study, we further explored the microarray dataset in order to add a potential upstream regulator of some of the enumerated signaling pathways in the early-onset CRC patients assessed in the Hong et al. study [3]. Specifically, we performed advanced statistical analysis to enhance the molecular understanding of the predictor gene set using text-mining and significant subpathways related to the early-onset CRC cases.

Our approach involves public text-mining [4] using a new statistical model that handles regulation (e.g., inhibition, activation) among biological entries, and performs a permutation test for subpathway identification of a given pathway. We first identified statistically significant subpathways related to the early onset CRCs from KEGG pathways [5] with the model, and subsequently used text-mining [4] to confirm literature associations among the predictor gene set and some representative significant subpathways.

Our proposed model suggests that early-onset CRC is involved in subcomponents of the focal adhesion pathway and the natural killer (NK) cell-mediated cytotoxicity pathway. The NK cell-mediated cytotoxicity pathway in particular hints at the presence of immune cells in the early-onset CRC patients, which implies paracrine communication between immune cells (e.g., NK cells, T cells, NK T cells) and various other cells [6]. In addition, our result indicates that the previously reported signaling pathways (Wnt, PI3K-AKT, MAPK) [3] are likely cascaded through their upstream focal adhesion kinase (FAK), [7] which belongs to the focal adhesion pathway. Therefore, FAK [7] may be a valuable therapeutic target candidate for the early-onset CRC predictor gene set diagnosis. Furthermore, our text-mining analysis of the 2 pathways along with the predictor gene set implied that some elements of the predictor gene set are involved in cell survival and epithelial-mesenchymal transition (EMT) [8], [9], [10] through the focal adhesion pathway and immunosuppression [8], [10], [11].

## Results

### Overview

**Figure 1:**
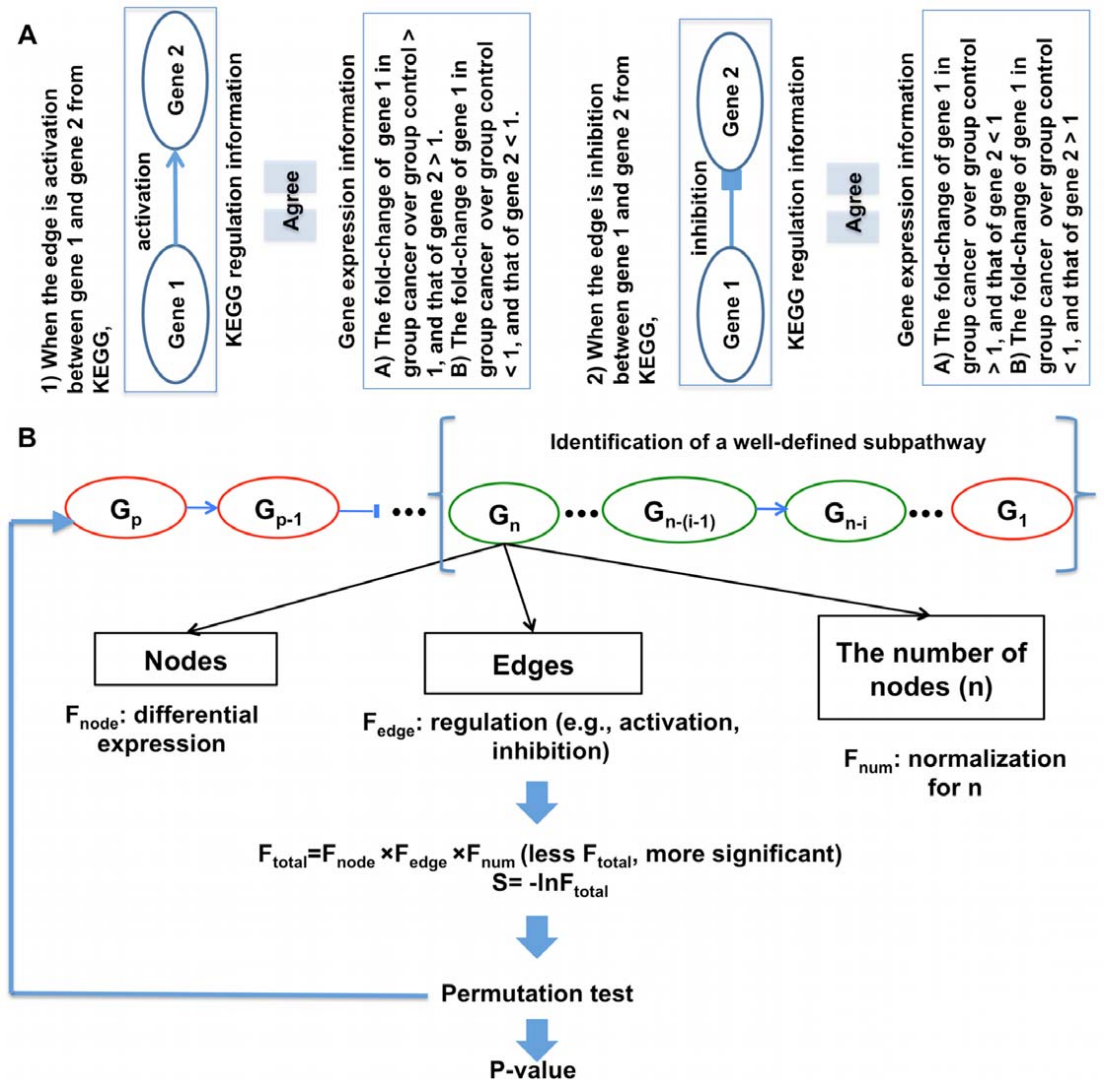
Rules and schematic diagram for the statistical model. **A.** Rules for matching an edge of two adjacent entities in KEGG pathways with their gene expression changes. Given an edge, gene 1 is called a source node of which the edge goes out, and gene 2 a sink node of which the edge comes in. **B.** Schematic diagram of the statistical model. Given a subpathway, the longest segment (well-defined subpathway) from the leaf node was identified. A statistic S for the well-defined subpathway was calculated. The null distribution of S was obtained via 1,000,000 sample label permutations and the p-value for the observed S was finally calculated (see *Materials and Methods* for details). Red ovals are up-regulated in the cancer patients, and green ones down-regulated.

The main concept of our statistical model was to pinpoint statistically significant subpathways whose expression (e.g., microarray) agreed with the regulation information (e.g., activation, inhibition) (Figure 1A) in the KEGG pathway database. Our approach is briefly described here.

**Figure 2:**
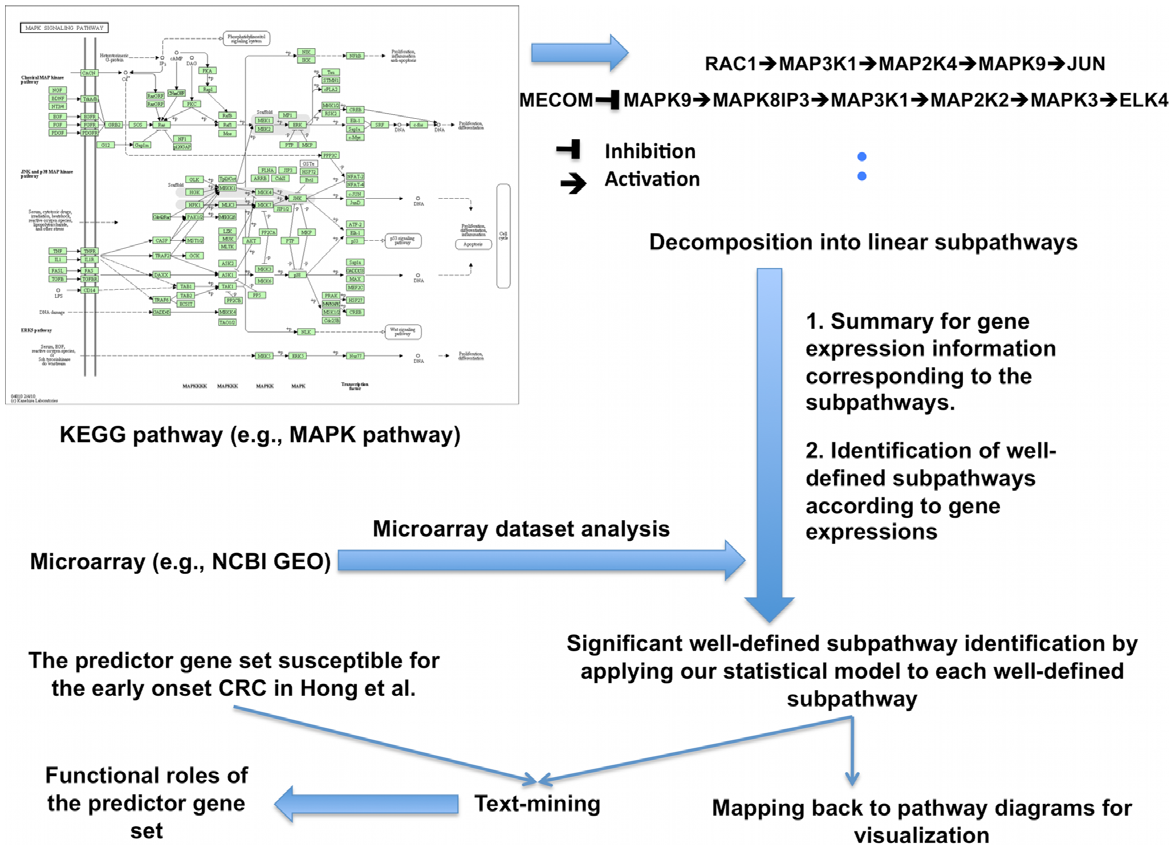
Overview of our study.

The non-metabolic KEGG pathways were reduced into linear subpathways, as described in the *Materials and Methods* (Figure 2). In this study, the term “linear subpathway” is used equivalent to “subpathway”. We then selected well-defined subpathways in which the gene expression agreed with the regulation information under the set rules (Figure 1A) as candidates for measuring their statistical significance (see *Materials and Methods*). A statistic *S* for each well-defined subpathway was calculated and its significance evaluated by computing the empirical *p*-value via sample label permutations (Figure 1B).

A total of 90 KEGG pathways were broken down into over 130 million extensive linear subpathways that considered all multiple gene assignments. Among these subpathways, 4,644 well-defined subpathways were identified and their significance evaluated via permutation tests. Subsequently, text-mining association analysis was performed for the selected significant well-defined subpathways; further discussion on their functional roles is provided hereafter.

### Significant well-defined subpathways

We performed multiple comparison tests by controlling the false discovery rate (FDR) [12]. The FDR *q*-values were computed using the *p*-values by performing 1 million permutation tests (Figure S1). The *p*-value that corresponded to an FDR of 5% was 0.01386, which yielded 1,289 significant well-defined subpathways. Since we opted not to provide detailed biological interpretation of all of these significant well-defined subpathways, we focused on examination of the top 30% of these well-defined subpathways to provide a more detailed biological description.

**Figure 3:**
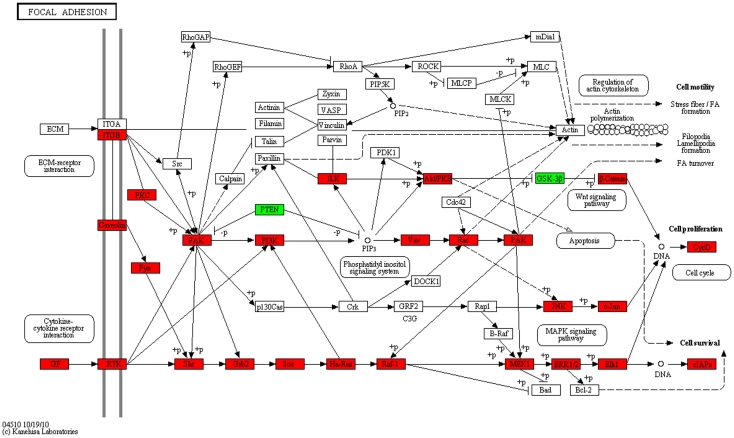
Mapping of the entries of the well-defined subpathways into the focal adhesion pathway. If the fold-change of the cancer patient group over the healthy control group is greater than one the gene is red, otherwise green. See Table 1 and Table S1 for detailed information.

**Figure 4:**
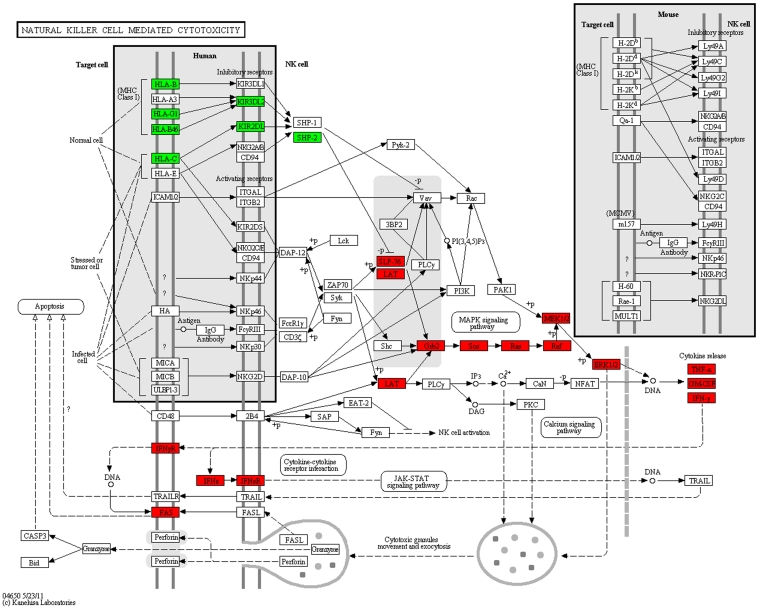
NK cell mediated cytotoxicity. Same description as Figure 3.

The majority of the selected subpathways we discuss belong to 6 KEGG pathways (Figure S2): Focal adhesion (KEGG hsa04510), Pathways in cancer (KEGG hsa05200), NK cell-mediated cytotoxicity (KEGG hsa04650), MAPK signaling pathway (KEGG hsa04010), Wnt signaling pathway (KEGG hsa04310), and Neutrophin signaling pathway (KEGG hsa04722). For the functional discussion and visualization, we mapped functionally interesting well-defined subpathways (Table S1) of the 6 KEGG pathways into KEGG pathway diagrams (Figures 3 and 4; Figures S3, S4, S5, and S6). In particular, we focused on 3 pathways (Focal adhesion, NK cell-mediated cytotoxicity, Pathways in cancer) that had not been explicitly mentioned in the previous Hong et al. study [3]. The gene entries of the well-defined subpathways included in the functional discussion and visualization of the 3 pathways are summarized in Table 1.

**Table 1:** The gene entries of the well-defined subpathways used for visualizing the three pathways diagrams (Figures 3 and 4, Figure S3).

Focal adhesion		NK cell cytotoxicity		Pathways in cancer	
AKT1	1.297	ARAF	4.631	ARAF	4.631
BIRC3 (c-IAP-2)	2.201	CSF2	1.879	BCR	1.241
CAV1	3.937	FAS	3.374	CCND1	1.180
CCND3	1.559	GRB2	1.613	CDK4	1.315
CTNNB1 (b-catenin)	2.562	HLA-B	0.795	CTNNB1 (b-catenin)	2.562
ELK1	2.593	HLA-C	0.655	DAPK1	0.438
FYN	4.286	HLA-G	0.693	DVL3	1.608
GRB2	1.613	HRAS	1.027	ETS1	1.805
GSK3B	0.735	IFNG	1.322	FGF13	5.486
HRAS	1.027	IFNGR1	2.086	FGFR1	2.138
IGF1	2.529	KIR2DL3	0.632	FIGF	3.458
ILK	1.467	KIR3DL2	0.721	FLT3	1.262
ITGB5	1.431	LAT	1.781	FLT3LG	3.022
JUN	4.179	LCP2	2.682	FOS	36.201
MAP2K1	1.162	MAP2K1	1.162	FZD10	6.256
MAPK1	2.425	MAPK1	2.425	GRB2	1.613
MAPK8	2.355	PTPN11	0.417	GSK3B	0.735
PAK3	2.780	SOS1	1.624	HRAS	1.027
PDGFRB	2.851	TNF	1.009	IGF1	2.529
PIK3CG	3.224	FASLG	2.096	IGF1R	2.299
PRKCA	3.061			IL8	4.276
PTEN	0.599			JUN	4.179
PTK2	2.151			KIT	1.430
RAC2	2.502			MAP2K1	1.162
RAF1	1.813			MAPK1	2.425
SHC3	1.838			MAPK8	2.355
SOS1	1.624			MMP2	3.031
VAV1	1.945			MYC	3.052
CYR61	80.630			NTRK1	1.225
				PDGFB	5.234
				PDGFRB	2.851
				RALGDS	1.478
				RET	2.212
				RHOA	4.286
				SOS1	1.624
				TCF7L1	2.735
				WNT3	3.147

The number represents the fold change of groups the CRC patient over the control. The genes CYR61 and FASLG were not reported in the statistical analysis but were added, considering their contexts in the pathways.

### Validation of the significant well-defined subpathways

We validated the entries in Table 1 by using an independent MedLine text-mining tool [4], PubGene. The purpose of this was to confirm whether the literature supported direct co-occurrences between the term “colorectal cancer” and the entries in Table 1. We found that 79% of the entries in Table 1 had direct interactions in the PubGene analysis (Table S2). Thus, we concluded that our model results provided a reasonable agreement with the literature examined.

### Pathways in cancer (hsa05200)

The KEGG pathway hsa05200 (pathways in cancer, Figure S3) is self-evident. Growth factor signaling, Wnt signaling, and MAPK signaling, which are located in the left part of Figure S3, were activated in the samples from CRC patients. The signals are common driving forces during carcinogenesis [8], [13]. Apparently normal mucosa in the CRC patients has an intrinsic potential for further transformation.

### Focal adhesion pathway (hsa04510)

Figure 3 shows the focal adhesion pathway. This result indicates that the bottom part of the pathway is highly involved with the CRC patients, and FAK (PTK2, Table 1) is not only a sink node from its upstream receptors but also a source node toward its downstream signaling transductions (Wnt, PI3K-AKT/PKB, and MAPK signals) for survival. PTEN (Table 1) [14], a tumor suppressor and antagonizer of the PI3K-AKT/PKB signaling pathway, was downregulated in the focal adhesion pathway in the analysis of the CRC patients' samples compared with that of the healthy controls' samples.

Hong et al. [3] suggested that the Wnt signaling pathway is involved in CRC patients. Our result regarding the focal adhesion pathway (Figure 3) supports the view that GSK-3β (GSK3B, Table 1) regulated by PI3K-AKT/PKB signaling of FAK downstream was downregulated in the CRC patients, and also that β-catenin (CTNNB1, Table 1) was highly expressed by downregulation of the Wnt signaling inhibitor GSK-3β in the CRC patients. Upon looking further into the information in Figure S5, we determined that gene expression of various activators and inhibitors related to Wnt signaling activation is consistent with the regulation flows. Another pathway, MAPK signaling (Figure S4) that was upregulated in the CRC patients is also located downstream of FAK (Figure 3).

Since the 3 activated signals (Wnt, PI3K-AKT/PKB, and MAPK signals) are located downstream of FAK belonging to the focal adhesion pathway, FAK [7], [15] may be a therapeutic target for the early-onset CRC predictor gene set diagnosis. Furthermore, because the crucial roles of Wnt, PI3K-AKT/PKB, and MAPK signaling shed light on EMT [9], there has been gradually increasing importance placed on FAK.

Interestingly, CYR61, which was included in the predictor gene set, is a ligand of ITGB5 (αVβ5 integrin, denoted as ITGB in Figure 3), according to the KEGG BRITE database [5]. Figure 3 shows that CYR61 is one of the far upstream cues that triggers FAK, implying that FAK subsequently activates 3 signals: Wnt, PI3K-AKT/PKB, and MAPK signaling. Recently, Wnt, PI3K-AKT/PKB, and MAPK signals were shown to be involved in EMT [9], and apparently normal mucosa in CRC patients could undergo phenotypical transformation by these 3 signals via the CYR61-FAK axis. In other words, some cells in the normal-appearing mucosa in the CRC patients may be close to atypical cells by utilizing EMT. We will describe the evidence of EMT in terms of gene expression level and explore any possible association between the predictor gene set and EMT in the *Discussion*.

Another finding relevant to the downstream region of the focal adhesion pathway is that the anti-apoptosis protein c-IAP (BIRC3, Table 1) [16], which is a negative regulator of caspases (e.g., CASP3, CASP8, CASP9), was also upregulated in the CRC patients. Thus, we further examined the various downstream c-IAPs (cellular inhibitor of apoptosis) in the focal adhesion pathway, where c-IAPs along with survivin (BIRC5) are also important anti-apoptotic proteins. Of note, it has also been shown that the c-IAPs and survivin also inhibit downstream caspases of both extrinsic and intrinsic apoptotic pathways [16]. We found that the majority of c-IAPs were upregulated in the CRC patients (Table 2). Thus, the focal adhesion pathway may confer inhibition of caspase activity upon the tumorigenesis of potential atypical cells in apparently normal mucosa.

**Table 2:** The gene expressions of c-IAPs, survivin (BIRC5) and XIAP (BIRC4).

Genes	Control^1^	Cancer^1^^,^ 2	Fold-change^3^
BIRC1	6.998	7.032	1.024
BIRC2	12.570	12.070	0.707
BIRC1///LOC648984///LOC653371	7.597	7.979	1.303
BIRC1///LOC653371	7.806	7.962	1.114
BIRC5	7.773	7.112	0.632
BIRC7	7.720	7.757	1.026
BIRC4	11.741	10.915	0.564
BIRC4BP	9.973	11.090	2.169
BIRC3	8.456	9.594	2.201
BIRC6	9.315	9.864	1.463

The majority of them except BIRC2, BIRC5, and BIRC4 were overexpressed in the cancer patients.

1 The value is the median of log2-scaled expressions of the group.

2 It is the normal-appearing mucosa in the CRC patients.

3 It represents fold-change of the cancer group over the control group.

### NK cell-mediated cytotoxicity pathway (hsa04650)

Our statistical analysis indicated significant agreement between the gene expression of the CRC patients and part of the immune pathway (hsa04650, Figure 4), which implies the presence of other immune cells as well as NK cells in the CRC patients' specimens.

FAS in the target cells of NK cells and its ligand (FASLG), which is produced by NK cells, were highly expressed in the CRC patients' samples. High FASLG expression in the CRC patients complies with previous clinical observations [6], [17] in which high FASLG expression was correlated with high incidences of metastases and poor survival in colorectal carcinoma patients and in other carcinoma patients.

In the apparently normal mucosa of the CRC patients, various target cells including potentially atypical cells may survive from FASLG-FAS death receptor signaling by escaping either extrinsic or intrinsic apoptotic signaling. In fact, the apoptotic signals were inhibited in the CRC patients because the c-IAPs [16] that inhibited caspases were upregulated in the CRC patients in terms of gene expression (Tables 1 and 2). Another possibility is that FASLG upregulation by target cells, including potentially atypical cells, might initiate fratricide and suicide among the immune cells with FAS beneficial for transformation of potentially atypical cells.

However, the existence of high interferon-gamma (IFN-γ) expression secreted by NK cells or immune cells in CRC patients remains controversial because NK cell cytotoxicity is traditionally believed to control immunosurveillance over cancer and atypical cells. Recently, a significant relationship between anti-tumor immunity and survival of cancer cells has been reported [6], [18]. Furthermore, IFN-γ is known to be involved in immunosurveillance against cancer cells, in multiple phenotypic effects on somatic cells (e.g., cell cycle progression, proliferation, cell differentiation, transformation), and in cancer cell escape [11], [18], [19], [20]. Thus, in the *Discussion*, we describe other roles of IFN-γ, especially in terms of the way cancer cells or potentially atypical cells in CRC patients could adjust the local immune system via immunosuppression in order to escape from immunosurveillance.

### Association among focal adhesion, NK cell-mediated cytotoxicity, and the early-onset CRC predictor gene set

**Figure 5:**
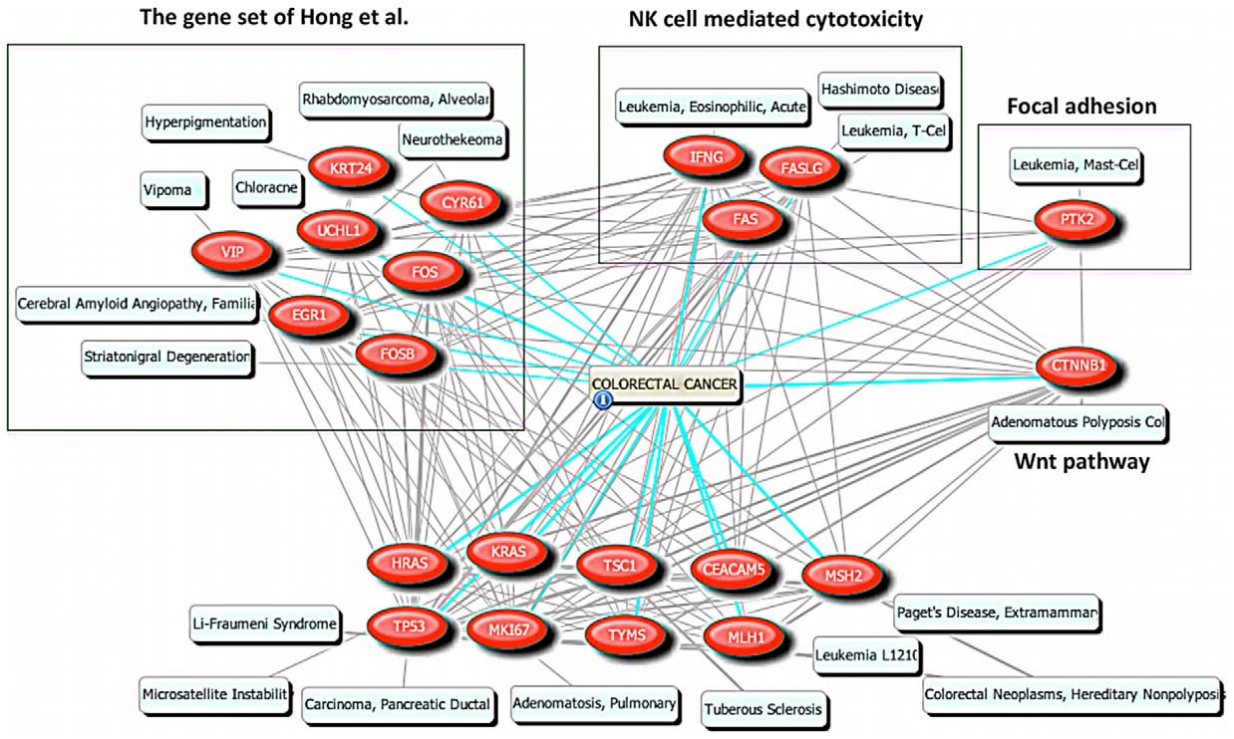
The association network of the susceptible gene set from Hong et al. and several representative genes from **Table 1**. The three green boxes represent the gene set (CYR61, FOS, FOSB, UCHL1, VIP, EGR1, KRT24), NK cell mediated cytotoxicity (IFNG, FAS, FASLG), and Focal adhesion (PTK2) from left to right. The options used in the network are described in Figure S7. The pale blue-filled boxes represent Mesh (www.nlm.nih.gov/mesh/) Diseases terms for the genes. It is noted that ITGB5 associations did not appear in the PubGene result.

As mentioned in the text above, Hong et al. [3] reported that early-onset susceptibility was attributed to the upregulated gene set called the “predictor gene set” in CRC patients that consists of *CYR61*, *EGR1*, *FOSB*, *FOS*, *VIP*, *UCHL1*, and *KRT24*. We inspected the associations among the genes listed in Table 1 and the predictor gene set with the text-mining tool, PubGene [4] (www.pubgene.org) (Figure S7). The input in the tool consisted of the predictor gene set, focal adhesion (FAK, ITGB5), and NK cell-mediated cytotoxicity (INFG, FAS, FASLG). Figure 5 shows an association network for the input genes in CRC. We already mentioned that β-catenin (CTNNB1, Wnt pathway in Figure 5) was regulated by FAK in focal adhesion. The predictor gene set, focal adhesion, and NK cell-mediated cytotoxicity were highly associated with each other in CRC.

### Comparison of our method with Gene Set Enrichment Analysis (GSEA) of the Hong et al. dataset

We compared the KEGG pathways containing significant well-defined subpathways identified by our method with those KEGG pathways obtained from the GSEA JAVA web start program (default options with 5,000 permutations). In our method, the significance level (*p*-value) was 0.05 for the cutoff of the well-defined subpathways. The same *p*-value of 0.05 was used for the GSEA method. Our method reported 1,966 significant well-defined subpathways that corresponded to 78 KEGG pathways. The GSEA program reported 2 broad types of significant pathway lists: 10 activated pathways and 30 repressed pathways in the CRC patients. The number of overlapping pathways between the 2 methods was 6, which is not surprising when considering the differences between 2 methods. Nevertheless, it is interesting that the 2 methods identified 6 common cancer-associated pathways.

To compare the 78 pathways identified by our method with the 40 pathways identified by GSEA, we used the cancer-related pathways reported by Vogelstein et al. [13] as a gold standard. That is, we inspected which method provided more pathways consistent with the cancer-related pathways identified by Vogelstein et al. The cancer-related pathways from the Vogelstein et al. study were manually mapped to their corresponding KEGG pathways because KEGG pathway identifiers corresponding to the cancer-related pathways were not mentioned explicitly in the study. We then inspected the overlapping pathways between the Vogelstein cancer-related KEGG pathways and those identified by the 2 methods. As shown in Table S3, our method provided more consistent results with the cancer-related pathways identified in Vogelstein et al. than did the GSEA method. Further details on this section are described in the Appendix S1.

### Comparison between the pathway substructure of the Hong et al. dataset and that of the other dataset

To determine how closely the pathway substructure of the Hong dataset overlaps with that of an additional colorectal dataset, we searched for an additional colorectal dataset from Gene Expression Omnibus (GEO). Although there are several datasets for CRC, it seems no datasets are available relating to a comparison of early-onset colorectal cancer patients with healthy controls, as is carried out in the Hong et al. study. Fortunately, we found the dataset GSE4183 [21], which compares various colorectal diseases (colorectal carcinoma, colorectal adenoma, inflammatory bowel diseases) with normal controls in a more general setting (www.ncbi.nlm.nih.gov/geo/query/acc.cgi?acc=GSE4183). From the GSE4183 dataset, we obtained normal, healthy controls (*n* = 8) and colorectal carcinomas (*n* = 15). The GSE4183 dataset was analyzed with our method, which revealed 3,669 well-defined subpathways (identified from ∼130 million subpathways) upon determining their significance based on 100,000 sample permutation tests. Furthermore, the comparison between the GSE4183 dataset and the dataset from Hong et al. (GSE4107) showed that 250 well-defined subpathways overlapped between the 2 datasets. To determine how well these 2 results coincided with each other, we also performed Fisher's exact test based on the randomization model. The *p*-value from the hypergeometric distribution was less than 2.2e-16, implying that the 2 results coincided well with each other. Thus, we conclude that our finding relevant to Hong's pathway substructure is well supported by our finding from the other independent dataset. Further details on this section are described in the Appendix S1.

## Discussion

Our novel analysis suggests the following results: 1) The subsets from focal adhesion, pathways in cancer, and NK cell-mediated cytotoxicity are highly involved in early-onset sporadic CRC patients; and 2) Surprisingly, the text-mining analysis suggested that the molecular function of the predictor gene set for early-onset sporadic CRCs is associated with focal adhesion and NK cell-mediated cytotoxicity. In the text below, we discuss the potential molecular mechanisms of this association in terms of immunosuppression and EMT.

### Immunosuppression

The recent literature [6], [11], [18], [19], [22], [23], [24] has set up a conceptual framework in which interactions between tumor and immunity are thought to help a number of cancer cells escape from immune-raiding by undergoing the following 3 phases in a linear or mixed manner: elimination (immunosurveillance), equilibrium (tumor dormancy), and escape (immunosuppression). In particular, cancer cell escape by immunosuppression [6], [8], [11], [19], [25], [26] has been extensively studied, and 2 types of immunosuppressive cells are thought to negatively regulate anti-tumor immune response: regulatory T cells (T_Reg_) and myeloid-derived suppressor cells (MDSCs) [8], [11], [27], [28]. We discuss below the roles of IFN-γ, other cytokines, and the predictor gene set in terms of these 2 types of immunosuppressive cells in early-onset CRC patients.

It has been demonstrated previously that IFN-γ can induce activation and expansion of MDSCs in colon cancer [28], and that activated MDSCs not only inhibit effector T cell activity/proliferation but also induce immunosuppressive CD4^+^CD25^+^Foxp3^+^ T_Reg_ cells from CD4^+^CD25^−^ T cells [6], [11], [28]. T_Reg_ cells, which also express CTLA-4, PD-1, and PD-L1 on their cell surfaces, positively regulate immunosuppressive cytokines interleukin (IL)-10 and tumor growth factor-beta (TGF-β), which can also induce T_Reg_ differentiation [6], [11], [29]. Since T_Reg_ cells are found in tumor infiltrating lymphocytes (TILs) in various cancers [6], [11], [29], the apparently normal mucosa in the CRC patients might have TILs present with immunosuppressive activity. ARG1 is also a key metabolic enzyme for MDSCs to negatively regulate lymphocyte functions by consuming or sequestering the amino acid arginine that is critical for T cell function. Thus, we inspected the gene expression levels of the examined genes (CD4, CD25, FOXP3, TGF-β, IFN-γ, IL-10, CTLA-4, PD-1, PD-L1, ARG1) in the CRC patients and confirmed they were all upregulated in the cancer patients (Table 3).

**Table 3:** The genes involved in immunosuppressive MDSCs and T_Reg_ cells in terms of immunosuppression.

Genes	Control^1^	Cancer^1^^,^ 2	Fold-change (Cancer/Control)
CD4	2.589	3.561	1.962
CD25 (IL2RA)	5.651	6.879	2.342
FOXP3	3.882	7.199	9.966
TGF-β (TGFB1)	7.841	9.229	2.617
IFN-γ (IFNG)	3.838	4.241	1.322
IL-10 (IL10)	4.784	5.887	2.148
CTLA-4 (CTLA)	5.460	7.398	3.832
PD-1 (PDCD1)	6.065	7.161	2.138
PD-L1 (CD274)	7.135	7.770	1.553
ARG1	3.079	4.004	1.899
CD11c (ITGAX)	6.553	7.747	2.288
CD11b (ITGAM)	5.757	6.854	2.139
CD33	4.567	5.470	1.870
CD34	7.162	8.798	3.108
FUT4	10.132	9.587	0.685
FUT9	2.141	3.585	2.721
Galectin-1 (LGALS1)	11.273	12.507	2.352
IL-6	5.976	7.342	2.578
IL-10	4.784	5.887	2.148
IDO (INDO, IDO1)	7.665	8.774	2.157

The majority of genes except FUT4 were up-regulated in the CRC patients.

1 The value is the median of the log2-scaled expressions of the group.

2 It is the normal-appearing mucosa in the CRC patients.

To provide gene expression-level evidence of the presence of MDSCs in CRC patients, we inspected (directly or indirectly) several MDSC surface markers: CD11c (ITGAX), CD11b (ITGAM), CD33, CD34, and CD15 [6]. Of note, we examined FUT4 and FUT9 instead of CD15 because CD15 is not a protein but an antigen synthesized by FUT4 and FUT9 [30]. We found that all of the markers except FUT4 were upregulated in the cancer patients (Table 3).

It has been shown previously that cancer cells expressing PD-L1 on their surface secrete immunosuppressive cytokines Galectin-1, IL-6, IL-10, and TGF-β, which can inhibit cytotoxic CD8^+^ T cells [11]. Furthermore, the cancer cells producing higher levels of indoleamine 2,3-dioxygenase (IDO) can prevent invasion of NK cells and effector T cells by depleting tryptophan essential for T cell function [6], [11]. In the present study, we confirmed a higher expression level of the examined genes (for PD-L1, Galectin-1, IL-6, IL-10, IDO, and TGF-β) in the cancer patients (Table 3).

The gene expression analysis shown in Table 3 suggests that immunosuppressive activity is highly observed in apparently normal mucosa. This finding could provide additional information about a “field change” [31], which refers to proliferation and anti-apoptotic activity in the apparently normal mucosa adjacent to tumor. In other words, anti-apoptosis of the field change could also benefit from immunosuppression by escaping the immune-raid.

To look further for functional refinement of the predictor gene set in terms of immunosuppression, we fed the predictor gene set into PubGene [4] with the MeSH (www.nlm.nih.gov/mesh) term “immunosuppression” (descriptor ID: D007165). The result (data not shown) obtained at the time of manuscript preparation indicated that 4 genes (*EGR1*, *FOS*, *UCHL1*, and *VIP*) have an association with immunosuppression according to the literature. Based on a review by Ganea et al., which was suggested by PubGene, VIP (a well-known immunoregulatory neuropeptide) inhibits the secretion of proinflammatory cytokines and induces T_Reg_ cells [32]. Other recent studies also support the immunosuppressive roles of VIP [33], [34] because VIP relieves collagen-induced arthritis and sarcoidosis by inducing CD4^+^CD25^+^Foxp3^+^ T_Reg_ cells from CD4^+^CD25^−^ T cells. VIP is also involved in immune privileges in the eye by inhibiting T lymphocyte activation and proliferation [35]. Thus, high VIP expression in CRC patients may pinpoint another major immunoregulatory cytokine in our analysis.

### EMT

We also inspected the expression level of EMT-related genes [9], [36], including matrix proteases, invasion molecules, epithelial/mesenchymal markers, and E-cadherin repressors. We found that the majority of them were upregulated in the cancer patients (Table 4). Therefore, the EMT [9] process can take place in the cancer patients, at least in terms of gene expression. This finding is unexpected in that atypical or precancerous cells could exist even in the normal appearing mucosa by cell morphology changes (e.g., EMT).

**Table 4:** The genes involved in EMT.

Functions	Genes	Control^1^	Cancer^1^^,^ 5	Fold-change^2^
Matrix proteases^3^	MMP2	5.265	6.865	3.031
	MMP3	6.463	6.371	0.938
	MMP9	9.667	11.131	2.758
	MMP10	3.641	5.181	2.908
	MMP11	4.321	5.659	2.529
	MMP13	3.995	4.250	1.194
	MMP14	6.383	7.259	1.836
	MMP16	5.137	6.288	2.220
Invasion molecules^3^	TWIST1	6.545	7.501	1.939
	SLUG (SNAI2)	8.798	9.373	1.489
	SDF-1 (CXCL12)	10.649	12.545	3.721
Epithelial markers^3^	E-cadherin (CDH1)	10.753	8.291	0.181
	TJP1	8.178	8.657	1.394
Mesenchymal markers^3^	N-cadherin (CDH2)	5.029	7.526	5.645
	Vimentin (VIM)	13.016	14.382	2.577
Transcriptional repressor of E-cadherin^4^	FOXC2	5.710	7.498	3.453
	SNAI1	5.762	7.511	3.362
	SLUG	8.798	9.373	1.489
	TWIST1	6.545	7.501	1.939
	ZEB2 (ZFHX1B)	6.015	7.734	3.293
	ZEB1 (TCF8)	6.731	8.026	2.453
	FOXC1	6.877	7.375	1.412
	GSC	4.048	3.781	0.831

The majority of the genes' expressions, except GSC, TJP1 and MMP3, indicate further malignant development of the early onset cancer group. Surprisingly, E-cadherin was down-regulated by more than 5 fold in the cancer patients.

1 The value is the median of log2-scaled expressions of the group.

2 The value is the fold-change of the cancer samples over the healthy controls.

3 The genes refer to Knutson et al. [36].

4 The genes refer to Polyak et al. [9].

5 It is the normal-appearing mucosa in the CRC patients.

To explore the potential roles of the predictor gene set, we input the predictor gene set into PubGene [4] with the MeSH term “Epithelial-Mesenchymal Transition” (descriptor ID: D058750). At the time of the manuscript preparation, 3 genes (*EGR1*, *FOS*, *CYR61*) out of the predictor gene set were found in the literature to have an association with EMT.

In particular, we paid attention to the gene *CYR61* because CYR61 is a ligand that can trigger a focal adhesion pathway. Monnier et al. [37] demonstrated that CYR61-αVβ5 integrin-induced metastasis was involved in the tumor bed effect after radiotherapy upon utilizing HCT116 CRC cell derivatives in hypoxic conditions. Additional recent studies on CYR61-driven development of cell motility in pancreatic ductal adenocarcinoma and in gastric epithelial cells [38], [39] indicate that CYR61 is one of the key molecules for EMT that could confer metastatic ability and cell motility to a primary tumor. Thus, CYR61 may be one of the driving molecules for enhancing EMT-related pathways (Wnt and PI3K/AKT signals) [9], [40] in early-onset CRC patients via the CYR61-FAK axis (Figure 3).

Another interesting finding we made upon examining the relationship between EMT and the predictor gene set was VIP, which was recently reported to induce EMT with the stimulation of matrix proteases matrix metalloproteinase (MMP)-2 and MMP-9 in prostate tumorigenesis [41]. We found that gene expression of these 2 proteases was indeed upregulated in the cancer patients (Table 4).

### Cytokines commonly involved in both EMT and immunosuppression

Because we found VIP is a cytokine involved in both EMT and immunosuppression, our finding implies paracrine signaling between immune cells and various target cells is involved in both processes. We also found an additional cytokine involved in the 2 processes, in that immunosuppressive TGF-β (TGFB1; Table S5) [22], [29], [36], [42] is a well-known EMT inducer [9], [43]. Indeed, we found that the majority of TGF-βs and their receptors were upregulated in the CRC patients.

### Conclusion

**Figure 6:**
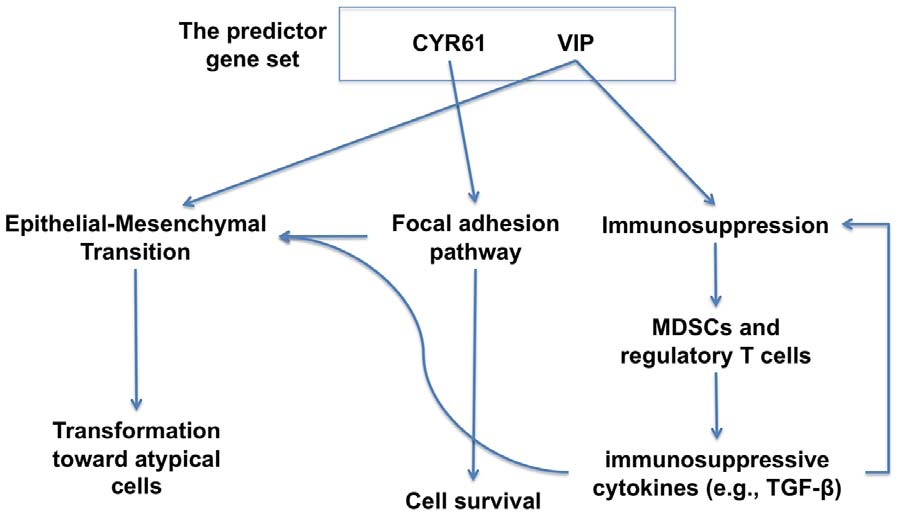
Summary of functional roles of the predictor gene set in terms of EMT and immunosuppression. The two elements (VIP, CYR61) of the predictor gene set can adjust the local immune system and induce malignant phenotype transformation via EMT.

Our gene expression data analysis suggests that at least 2 entries (*VIP*, *CYR61*) of the predictor gene set are functionally involved in phenotypical EMT induction by focal adhesion downstream (Wnt, PI3K/AKT, MAPK) and immunosuppression (Figure 6). The involvement of EMT in the apparently normal mucosa of the CRC patients suggests that a subpopulation of cells in the mucosa have experienced intrinsic transformation toward atypical or cancerous phenotypes. Furthermore, potential atypical cells may survive against immune cells by utilizing immunosuppressive cytokines (e.g., VIP, TGF-β). Promotion of such an intrinsic survival environment in the apparently normal mucosa is closely aligned with the clinical observation of a field change [31], which refers to proliferation and anti-apoptotic activity in apparently normal mucosa adjacent to tumor. In the process of immunosuppression, the immunosuppressive cytokines VIP (a member of the predictor gene set) and TGF-β may be highly involved in the dynamics between potential atypical cells and immune cells via paracrine signaling (Figure 6). Our study suggests the co-occurrence of EMT and immunosuppression [36] even in normal-appearing mucosa in early-onset CRC patients. Finally, our biological suggestion needs to be validated experimentally in future studies on early-onset CRC in terms of dedifferentiation or differentiation, which is underscored in EMT and immunosuppression.

## Materials and Methods

### Data

Gene expression data for early-onset CRCs were downloaded from NCBI GEO (www.ncbi.nlm.nih.gov/geo/); the dataset identifier is GSE4107 [3]. This dataset consists of data for 12 CRC patients and 10 healthy controls. Normal-appearing mucosa adjacent to tumor had been obtained from the CRC patients and normal mucosa obtained from the healthy controls. The predictor gene set was derived from comparison between the normal-appearing mucosa from the cancer patients and the normal mucosa from the controls. Of note, the patients did not have FAP or HNPCC. We obtained prior regulation information from KEGG [5].

### Decomposition of the KEGG pathways into linear subpathways

For simplicity, all the pathways of interest were divided into linear subpathways by modifying the CPAN Paths::Graph library (search.cpan.org/∼cavasquez/Paths-Graph/Graph.pm) (Figure 2). The linear subpathway is a sequence of linearly connected gene entities from root node to leaf node. The root nodes are generally membrane receptors, their ligands, and so on. The leaf nodes are usually transcription factors and signaling initiators toward the other pathways. We extracted as many linear subpathways as possible, considering multiple gene assignments of each node.

### Rules for gene expression and edge information of KEGG

Our goal was to identify subpathways in which gene expression agreed with prior regulation information (e.g., activation, inhibition) in KEGG pathways. The gene regulation among the entries in KEGG pathways was considered to be prior knowledge. Edge types in KEGG represent regulations between the connected entities. We simplified the edges into only 2 types: activation and repression. We also assumed rules for matching an edge type of 2 adjacent entities to their gene expression changes (Figure 1A) [44]. Given a subpathway, we identified the longest consecutive segment beginning from its leaf node; the segment had to satisfy the assumed rules. The segment is referred to as a “well-defined subpathway” in terms of gene expression data and prior knowledge (Figure 1B). Further mathematical representation is also described below detailing how we obtained the well-defined subpathway.

**Figure 7:**
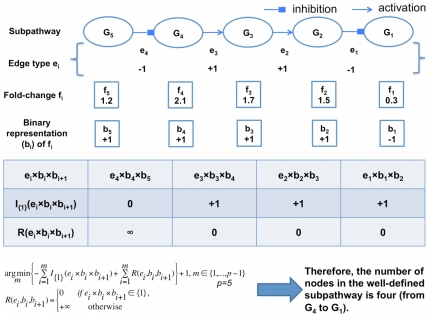
The example of determination of the well-defined subpathway from a subpathway.

Given a subpathway with the number of nodes (genes) *p*, the leaf node was set to G_1_ and the root node to G_p_. The node G_i_ had its binary representation (*b_i_*) of a fold-change (*f_i_*) for cancer over control that was obtained from gene expression data. If *f_i_*>1, then *b_i_* was +1, otherwise it was −1. The prior edge type *e_i_* between the source node G_i+1_ and the sink node G_i_ was either +1 (activation) or −1 (repression) (Figures 1B and 7). The expression *e_i_*×*b_i_*×*b_i+1_* should have been equal to 1 if the expression matched with the regulations under the rule. Again, edge information *e_i_* was derived from the prior knowledge from KEGG, and *f_i_* and *b_i_* were derived from gene expression data. In summary, the number of nodes (*n*) of a well-defined subpathway was defined as follows:
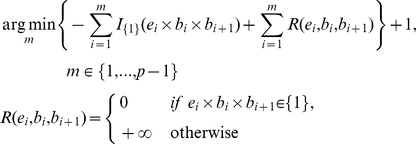
The function *I*(·) represents the indicator function and the function *R*(·) is a penalizing term that prevents probing progression from the leaf node to the root node when prior edge information and expression data did not agree with the rules. Figure 7 shows an example of identification of the well-defined subpathway using the previous equation, given a subpathway. It is noted that mathematical notation is also used throughout the manuscript.

### Statistical analysis

In this section, we describe a statistical model to determine significance for the well-defined subpathways. We conceptually divided the well-defined subpathway into 3 components referred to as node score (F_node_), edge score (F_edge_), and score for the number of nodes (F_num_), ultimately in order to define a total score (F_total_) of the well-defined subpathway. We designed F_total_ such that the more differentially expressed genes agreed with the rule (Figure 1A) in the well-defined subpathway, the less F_total_ was equal to. F_node_ represents the differential expression of the entries, F_edge_ represents the regulation among the adjacent entries, and F_num_ represents length normalization. F_num_, a normalization factor for *n* (the number of nodes in the well-defined subpathway), is necessary because the longer *n* was, the less F_total_ was when different well-defined subpathways were compared. In the text below, we describe the biological rationale and mathematical representation for the scores.

Before providing further explanation, we must define the terms source node and sink node. That is, given edge e_n−i_, the upstream node G_n−(i−1)_ is called a source node, whereas the downstream node G_n−i_ is called a sink node (Figure 1A, B).

Typical expression analysis schemes focus only on highly differentially expressed genes under a certain cutoff (e.g., *p*-value), but it is important to consider that signaling proteins of an activated or repressed pathway involved in phenotype differences might not be changed drastically at the expression-level [45]. In other words, employing strict cutoff usage in gene expression data involves difficulties in uncovering signal cascading flows because some entries within the signal cascading flows could be missed under that cutoff. In contrast, F_node_ does not filter out low differential expression with an arbitrary condition because the *p*-values of all the entries within the well-defined subpathway are considered.
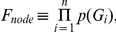
where *n* is the number of nodes in the well-defined subpathway and *p*(·) is a *p*-value of a gene in a two-sample *t*-test between the cancer and control groups. Therefore, the node score contains both high and low differential expressions without a strict cutoff.

F_edge_ reflects edge information (e.g., activation, inhibition) between 2 adjacent entries and derives from a joint distribution of activities of a source node and its corresponding sink node. The basic idea of F_edge_ is that, given a source node activity of edge e_n−i_, its corresponding sink node activity is expected to be highly dys-regulated, which indicates a rare event. Therefore, F_edge_ follows, by nature, the first-order Markov chain property in which a current event depends only on its predecessor because we assume that G_n−i_ is only regulated by its direct upstream source node G_n−(i−1)_ of edge e_n−i_.

In Figure 1A, we used fold-changes to determine whether the edge information complied with gene expression. The activities around the edge were thus measured from fold-changes in cancer over control, and the activity was simply transformed into a log_2_ scale for better normality. In other words, log_2_-transformed fold-changes of the 2 adjacent nodes were used as a measure of edge information, and the joint distribution of a source node and its sink node was calculated.

According to the first-order Markov chain property, we can simply define F_edge_ as the products of conditional probabilities log_2_(f_n−i_)|log_2_(f_n−(i−1)_) from i = 1 to *n*−1 multiplied by a prior Pr(log_2_(*f*_n_)). The conditional probabilities can be derived from the joint distribution of the pair (log_2_(fold-change of the source node), log_2_(fold-change of the sink node)), which is assumed to be a bivariate normal distribution.
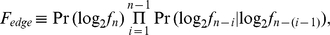
where *n* is the number of nodes in the well-defined subpathway.

To determine the joint distribution of the pair (log_2_(fold-change of the source node), log_2_ (fold-change of the sink node)), we extracted all the edges from the KEGG XML files (KGML files) and obtained the source nodes and their corresponding sink nodes from the edges. The log_2_-transformed fold-changes (e.g., a pair (log_2_ *f_n_*_−(*i*−1)_, log_2_ *f_n_*_−*i*_)) of the cancer group over the control group for the pair source node and sink node were obtained from the microarrays.
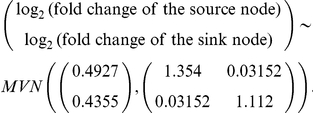
Prior probability (e.g., Pr(log_2_ *f_n_*)) of the log_2_-transformed fold-change of a gene was assumed to be a univariate normal distribution, which is obtained from the log_2_-transformed fold-changes of all the entries belonging to the KEGG pathways.

F_num_ was derived from a random graph model. We used an *R* statistical package library “igraph” [46] to make 1,000 simulated random graphs with the number of nodes set to 200 and the number of edges to 100. We reduced the random graphs into all linearly connected paths from roots to leaves, and the distribution of the number of edges for the paths was calculated (Figure S8). F_num_ was borrowed from the distribution of the number of edges from the random graphs. Subsequently, *n* (the number of connected nodes in the well-defined subpathway) was considered as equal to 1 plus the number of edges in the well-defined subpathway.

Finally, we defined F_total_ as follows:
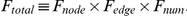
For computational simplicity, we used its minus natural logarithm of F_total_ as a statistic instead: 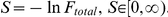


### Permutation

We obtained the null distribution of *S* by generating 1,000,000 permuted samples. Each permuted sample was generated by shuffling the sample labels in the microarrays. For each permutation, we applied the rules to the original subpathways in order to redefine their well-defined subpathways, and then performed the same procedures discussed above. The *p*-value was obtained empirically by computing Pr(*S*≥*s*), where *s* was the observed value in the original gene expression data.

We also provide detailed information of all 4,644 well-defined subpathways in Table S4 (xls format), including their *p*-values, FDR *q*-values, and regulation information.

## Supporting Information

Figure S1 **Statistic S, p-value and multiple comparison correction.** **A.** S versus −log_10_(p-value) in the 4,644 well-defined subpathways. The x-axis represents −1og_10_(p-value) and the y-axis S. **B.** −log_10_(p-value) versus FDR q-value. The x-axis is FDR q-value and the y axis −log_10_(p-value). The FDR q-values as well as p-values were summarized in Table S4 (see the sixth and eighth columns in Table S4).
(DOC)

Figure S2 **KEGG pathways containing the top 30% well-defined subpathways.** The x-axis represents the number of the significant well-defined subpathways corresponding to the KEGG pathway.
(DOC)

Figure S3 **Pathways in cancer (KEGG hsa05200).** Red boxes are activated in the CRC patients over the healthy controls. Green boxes are down-regulated in the CRC patients.
(DOC)

Figure S4 **MAPK signaling pathway (KEGG hsa04010).** Red boxes are activated in the CRC patients over the healthy controls. Green boxes are down-regulated in the CRC patients.
(DOC)

Figure S5 **Wnt signaling pathway (KEGG hsa04310).** Red boxes are activated in the CRC patients over the healthy controls. Green boxes are down-regulated in the CRC patients.
(DOC)

Figure S6 **Neutrophin signaling pathway (KEGG hsa04722).** Red boxes are activated in the CRC patients over the healthy controls. Green boxes are down-regulated in the CRC patients.
(DOC)

Figure S7 **The input item options used in** **Figure 5** **.** The item “Gene/Protein” in the PubGene input webpage is CYR61, FOS, FOSB, UCHL1, VIP, EGR1, KRT24, PTK2, ITGB5, IFNG, FAS, and FASLG. The item “Biological term” in the webpage is colorectal cancer.
(DOC)

Figure S8 **Distribution of the number of edges in the linearly connected paths based on the 1,000 simulated random graphs.** The x-axis represents the number of the edges, and the y-axis probability.
(DOC)

Table S1 **The numeric identifiers of the well-defined subpathways used for the functional discussion and visualization of the six KEGG pathways.** The number indicates column “No.” in Table S4 (xls format). Readers see all the information of significance, regulation flow, fold-change and so on from Table S4.
(DOC)

Table S2 **We fed the entries in** **Table 1** **into PubGene in order to validate literature-based associations between our result and the term “colorectal cancer”.** The listed genes have no direct co-occurrence with the term “colorectal cancer” according to PubGene. The majority (79%) of the entries in Table 1 have publication-based evidences. It is noted that CYR61 and FASLG in Table 1 were not included in the PubGene validation analysis because the two genes were not reported in our statistical analysis.
(DOC)

Table S3 **Comparison with our method and GSEA.** We set the Vogelstein cancer-related pathways [13] (first column) as a gold standard. We inspected overlap between the gold standard and each method result. As a result, our method performed better than GSEA. The second column represents KEGG pathways corresponding to the first column. (O: overlap, X: no overlap)
(DOC)

Table S4 **Detailed information of all the 4,644 well-defined subpathways.** No.: numeric identifier for the well-defined subpathway, KEGG: its corresponding KEGG pathway identifier, Title: KEGG pathway name, WellDefinedSubpathwayWithFoldChange: signaling flow of the well-defined subpathway with fold-change of the cancer patients over the healthy control, NumNodes: the number of entries, P-value: nominal p-value, S: our statistic, FDR (q-value): adjusted p-value, −log10(P-value): minus logarithm of p-value with base 10.
(XLS)

Table S5 **The expressions of TGF-βs and their receptors were summarized.** Majority of the genes were up-regulated in the cancer except TGFBR1.
(DOC)

Appendix S1 **The additional analysis for GSEA comparison and independent dataset validation.**
(DOC)
