# Supplementary figures and images for: Pathway-Based Evaluation in Early Onset Colorectal Cancer Suggests Focal Adhesion and Immunosuppression along with Epithelial-Mesenchymal Transition
Source: PLoS One. 2012 Apr 9;7(4):e31685. doi: 10.1371/journal.pone.0031685 (PMC3322137; doi:10.1371/journal.pone.0031685)

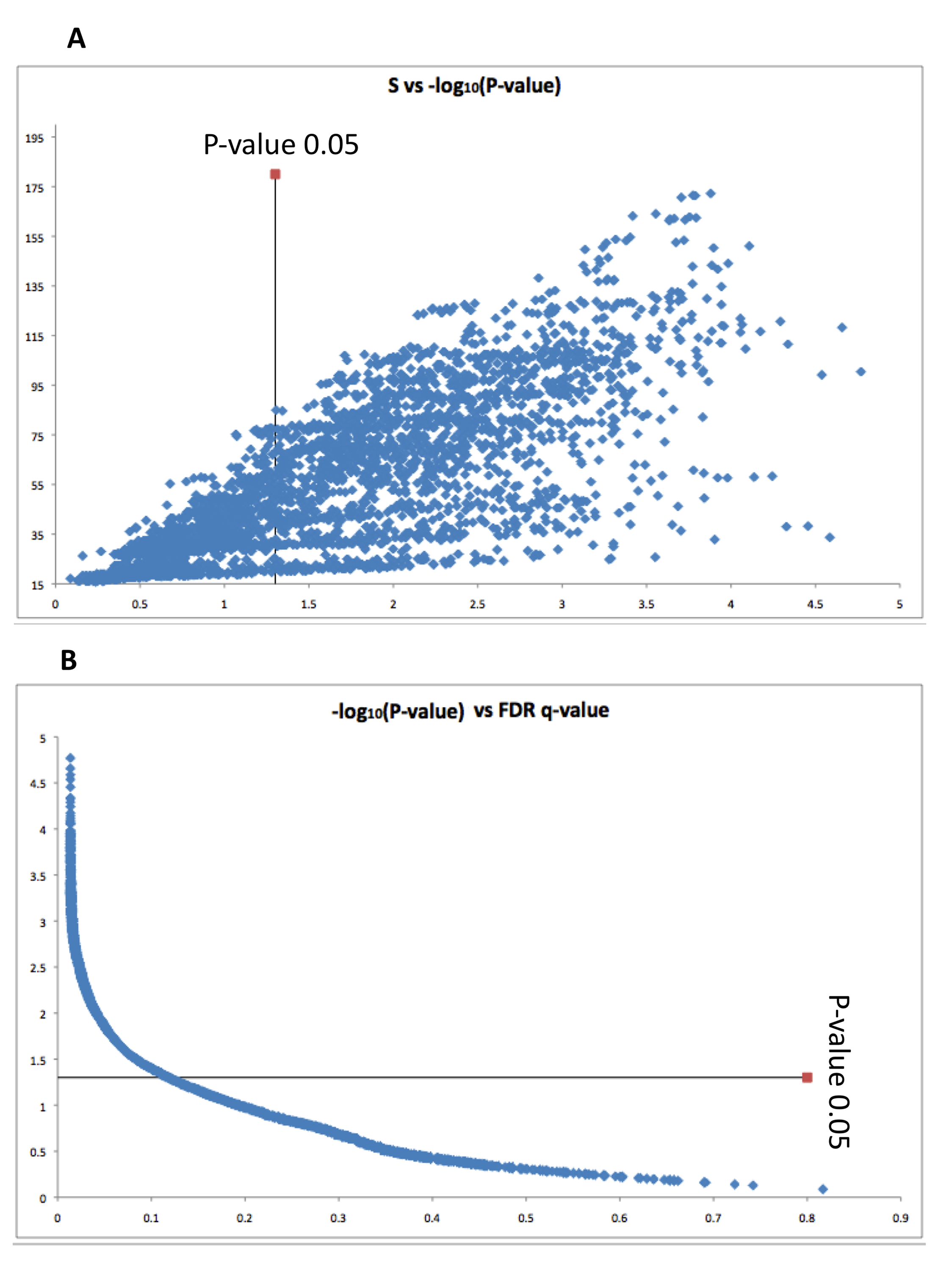

Supplement: Figure S1 — Statistic S, p-value and multiple comparison correction. A. S versus −log10(p-value) in the 4,644 well-defined subpathways. The x-axis represents −1og10(p-value) and the y-axis S. B. −log10(p-value) versus FDR q-value. The x-axis is FDR q-value and the y axis −log10(p-value). The FDR q-values as well as p-values were summarized in Table S4 (see the sixth and eighth columns in Table S4). (DOC) [file pone.0031685.s001.doc]

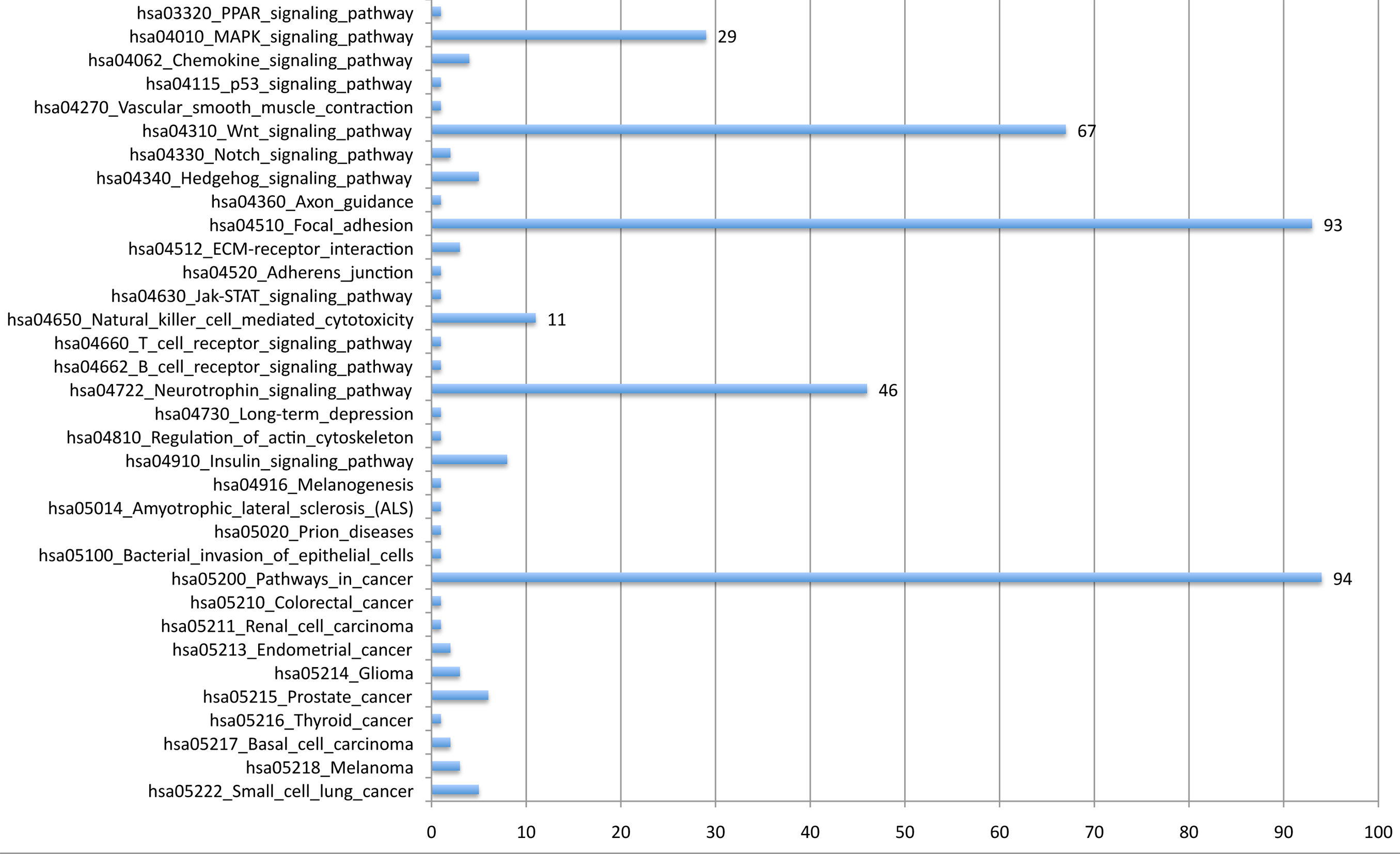

Supplement: Figure S2 — KEGG pathways containing the top 30% well-defined subpathways. The x-axis represents the number of the significant well-defined subpathways corresponding to the KEGG pathway. (DOC) [file pone.0031685.s002.doc]

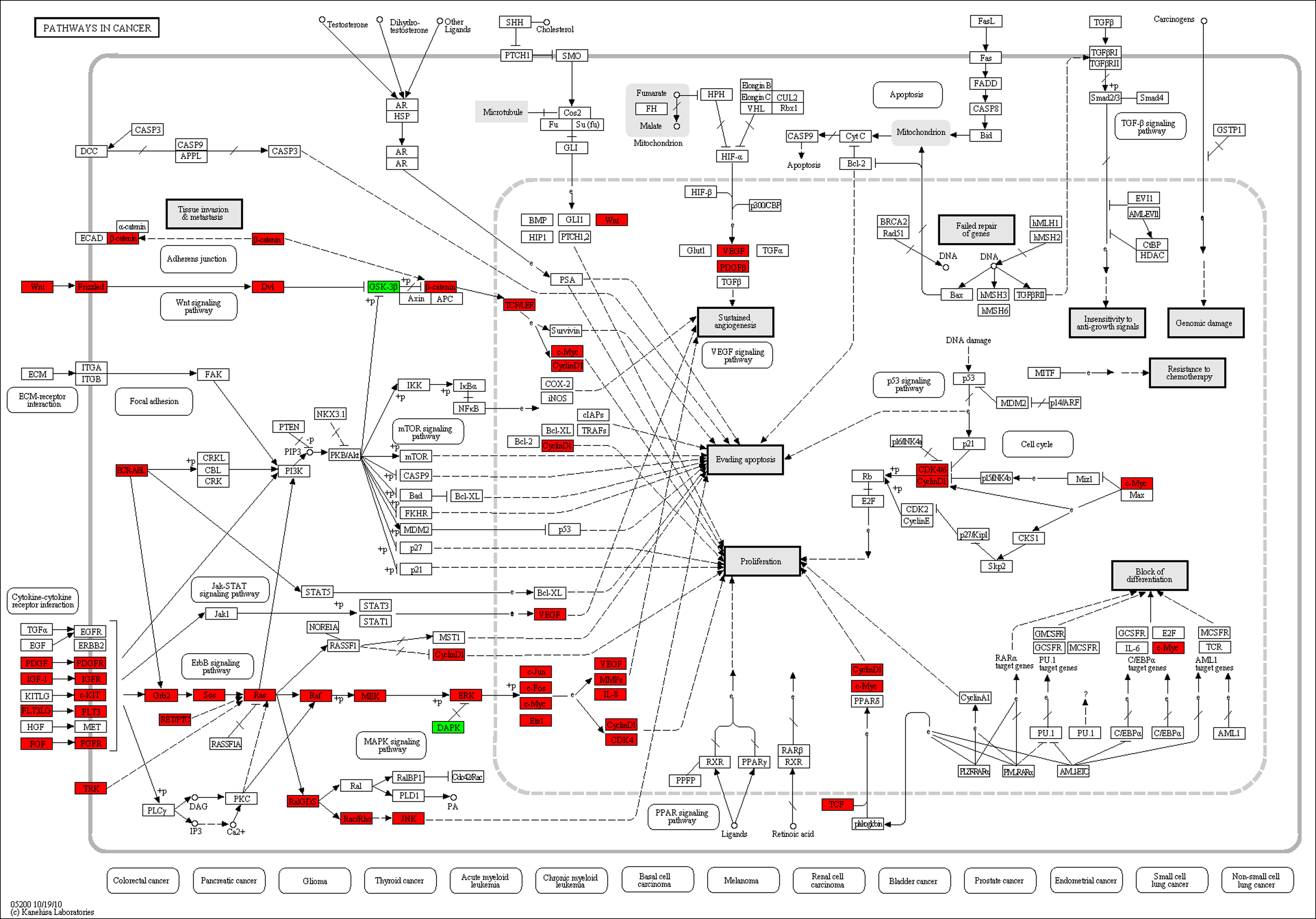

Supplement: Figure S3 — Pathways in cancer (KEGG hsa05200). Red boxes are activated in the CRC patients over the healthy controls. Green boxes are down-regulated in the CRC patients. (DOC) [file pone.0031685.s003.doc]

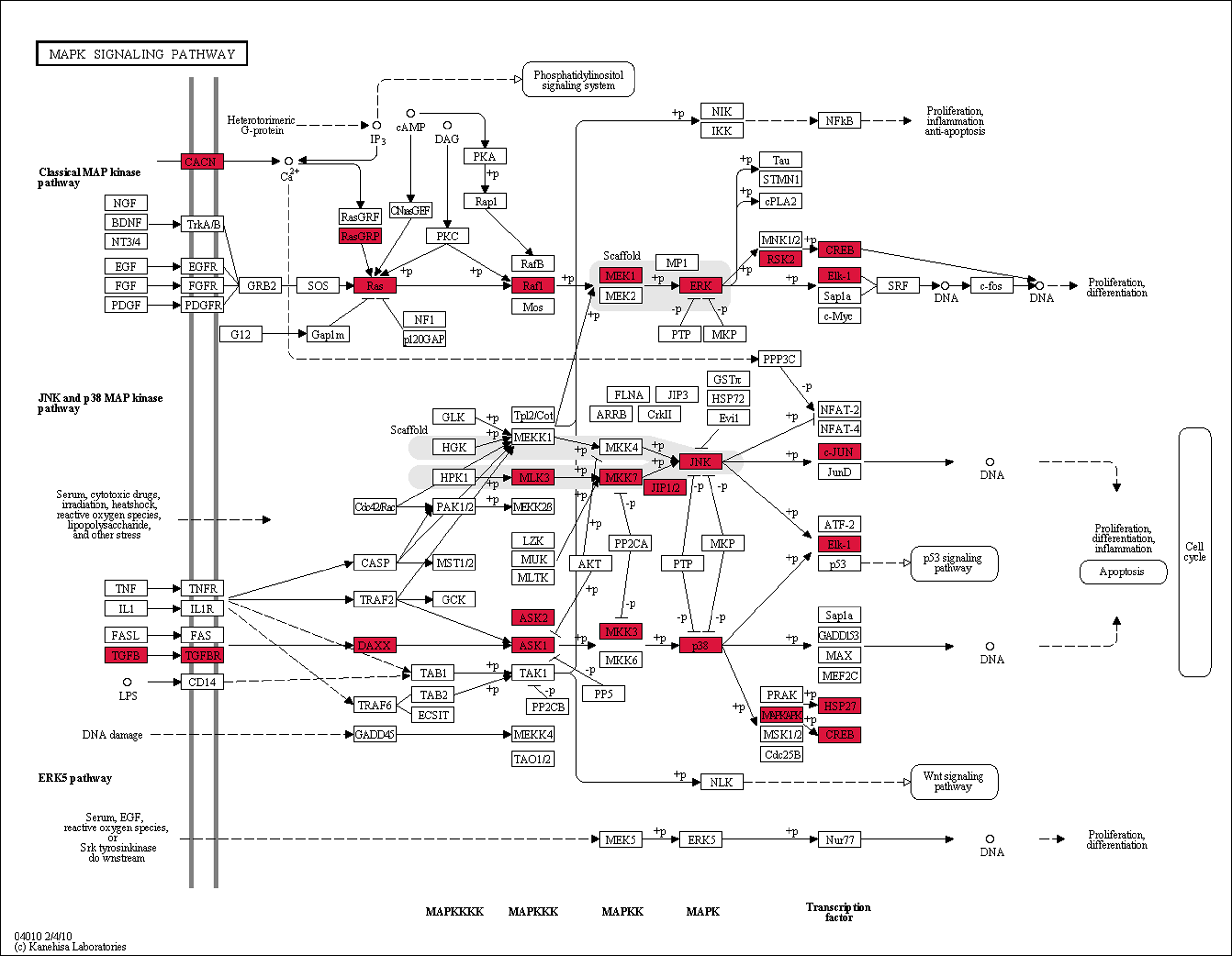

Supplement: Figure S4 — MAPK signaling pathway (KEGG hsa04010). Red boxes are activated in the CRC patients over the healthy controls. Green boxes are down-regulated in the CRC patients. (DOC) [file pone.0031685.s004.doc]

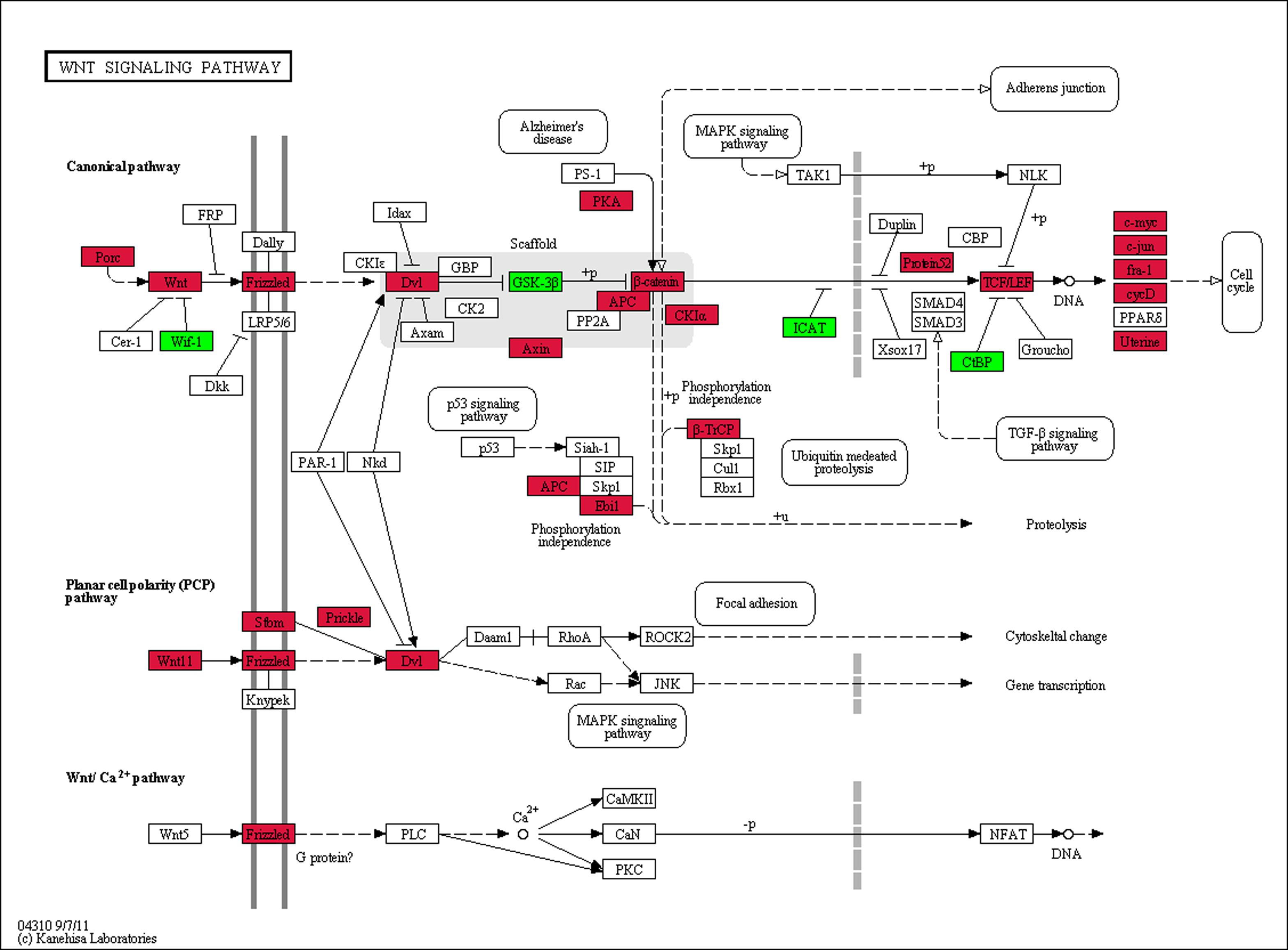

Supplement: Figure S5 — Wnt signaling pathway (KEGG hsa04310). Red boxes are activated in the CRC patients over the healthy controls. Green boxes are down-regulated in the CRC patients. (DOC) [file pone.0031685.s005.doc]

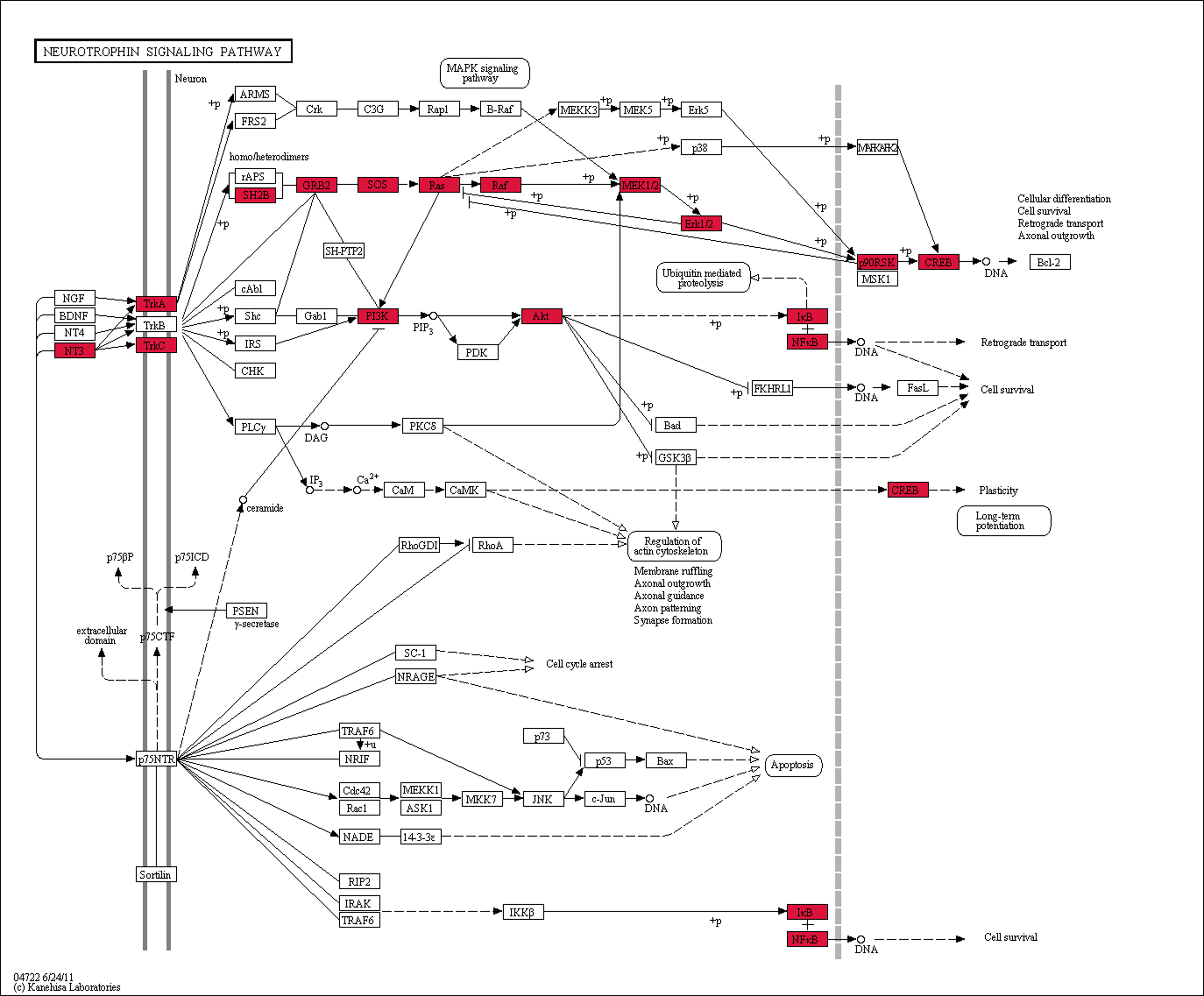

Supplement: Figure S6 — Neutrophin signaling pathway (KEGG hsa04722). Red boxes are activated in the CRC patients over the healthy controls. Green boxes are down-regulated in the CRC patients. (DOC) [file pone.0031685.s006.doc]

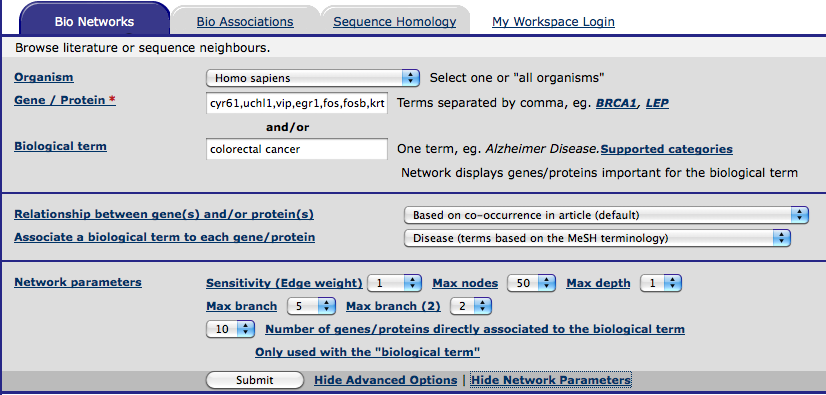

Supplement: Figure S7 — The input item options used in Figure 5 . The item “Gene/Protein” in the PubGene input webpage is CYR61, FOS, FOSB, UCHL1, VIP, EGR1, KRT24, PTK2, ITGB5, IFNG, FAS, and FASLG. The item “Biological term” in the webpage is colorectal cancer. (DOC) [file pone.0031685.s007.doc]

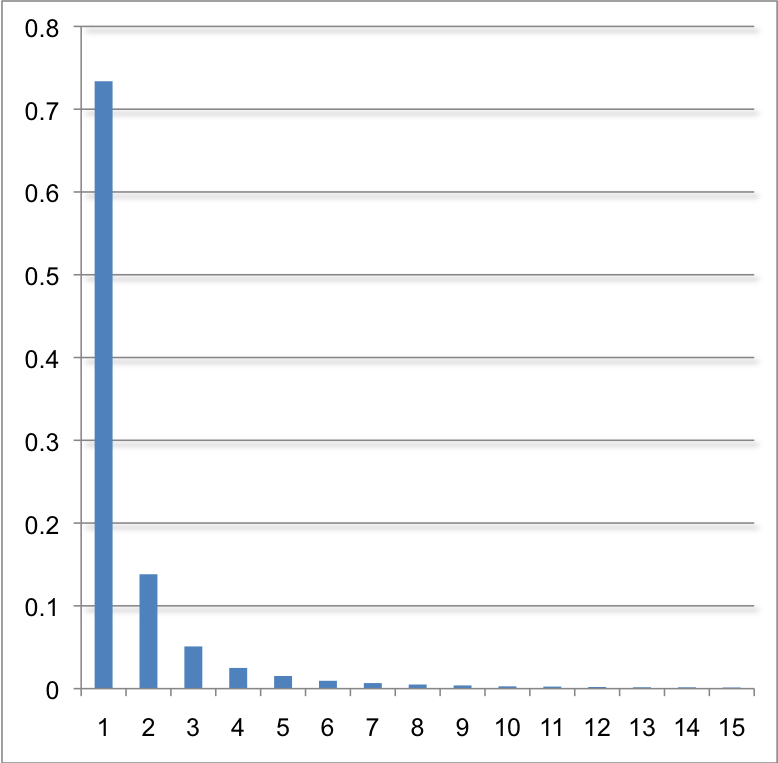

Supplement: Figure S8 — Distribution of the number of edges in the linearly connected paths based on the 1,000 simulated random graphs. The x-axis represents the number of the edges, and the y-axis probability. (DOC) [file pone.0031685.s008.doc]
