# Supplementary material for: Pathway-Based Evaluation in Early Onset Colorectal Cancer Suggests Focal Adhesion and Immunosuppression along with Epithelial-Mesenchymal Transition
Source: PLoS One. 2012 Apr 9;7(4):e31685. doi: 10.1371/journal.pone.0031685 (PMC3322137; doi:10.1371/journal.pone.0031685)
Supplement: Table S1 — The numeric identifiers of the well-defined subpathways used for the functional discussion and visualization of the six KEGG pathways. The number indicates column “No.” in Table S4 (xls format). Readers see all the information of significance, regulation flow, fold-change and so on from Table S4. (DOC) [file pone.0031685.s009.doc]

| Pathway | The well-defined subpathways for the pathway diagram |
| --- | --- |
| HSA04510 | 12,17,21,25,28,29,31,43,45,47,48,49,50,51,52,53,54,56,57,59,62,66,68,70,71,74,76,78,79,80,89,90,92,96 |
| HSA04650 | 146,180,201,229,235,251,322,334,341,369,376 |
| HSA05200 | 2,6,8,10,13,14,15,16,19,20,22,24,30,33,34,38,39,41,44,55,65,69,75,83,85,86,87,88,91,93,95,97,98,102,104,105,106,113,115,117,118,128,129,134,140,143,144,145,148,158,160,172,175,186,188,190,199,204,205,212,222,225,230,245,246,248,249,250,255,261,263,271,273,275,276,278,279,281,282,295,298,303,304,305,310,327,338,344,346,347,352,370,380,388 |
| HSA04010 | 9,35,36,72,103,107,132,161,170 |
| HSA04310 | 11,18,23,26,32,40,42,46,58,60,67,81,82,84,108,110,111,116,119,125,126,127,135,139,141,147,163,166,173,181,185,189,191,192 |
| HSA04722 | 64,77,100 |

Table S1.
