# Supplementary material for: Pathway-Based Evaluation in Early Onset Colorectal Cancer Suggests Focal Adhesion and Immunosuppression along with Epithelial-Mesenchymal Transition
Source: PLoS One. 2012 Apr 9;7(4):e31685. doi: 10.1371/journal.pone.0031685 (PMC3322137; doi:10.1371/journal.pone.0031685)
Supplement: Table S2 — We fed the entries in Table 1 into PubGene in order to validate literature-based associations between our result and the term “colorectal cancer”. The listed genes have no direct co-occurrence with the term “colorectal cancer” according to PubGene. The majority (79%) of the entries in Table 1 have publication-based evidences. It is noted that CYR61 and FASLG in Table 1 were not included in the PubGene validation analysis because the two genes were not reported in our statistical analysis. (DOC) [file pone.0031685.s010.doc]

| Pathway | Genes that are not co-occurred with the term "colorectal cancer" |
| --- | --- |
| Focal adhesion | VAV1,SOS1,ELK1,RAC2 ,ITGB5,MAPK8,SHC3 |
| NK cell cytotoxicity | SOS1,LAT,LCP2,KIR3DL2 |
| Pathways in cancer | DVL3,FLT3LG,TCF7L1,SOS1,RALGDS,MAPK8,WNT3 |

Table S2.
