# Supplementary material for: Pathway-Based Evaluation in Early Onset Colorectal Cancer Suggests Focal Adhesion and Immunosuppression along with Epithelial-Mesenchymal Transition
Source: PLoS One. 2012 Apr 9;7(4):e31685. doi: 10.1371/journal.pone.0031685 (PMC3322137; doi:10.1371/journal.pone.0031685)
Supplement: Table S3 — Comparison with our method and GSEA. We set the Vogelstein cancer-related pathways [13] (first column) as a gold standard. We inspected overlap between the gold standard and each method result. As a result, our method performed better than GSEA. The second column represents KEGG pathways corresponding to the first column. (O: overlap, X: no overlap) (DOC) [file pone.0031685.s011.doc]

| **Vogelstein** | **Corresponding KEGG pathway** | | **Our method** | **GSEA** |
| --- | --- | --- | --- | --- |
| HIF1 | HSA04150 | mTOR signaling pathway | X | O |
|  | HSA05200 | Pathways in cancer | O | X |
|  | HSA05211 | Renal cell carcinoma | O | X |
| P53 | HSA04115 | P53 signaling pathway | O | X |
| RB (cell-cycle) | HSA04110 | Cell cycle | O | X |
| Apoptosis | HSA04210 | Apoptosis | O | X |
| GLI | HSA04340 | Hedgehog signaling pathway | O | X |
| APC | HSA04310 | Wnt signaling pathway | O | X |
| RTK | HSA04012 | ERBB signaling pathway | O | X |
|  | HSA05200 | Pahtways in cancer | O | X |
| SMAD | HSA04350 | TGF-Beta signaling pathway | O | X |
| PI3K | HSA04012 | ERBB signaling pathway | O | X |
|  | HSA05200 | Pathways in cancer | O | X |
|  | HSA04150 | mTOR signaling pathway | X | O |
|  | HSA04010 | MAPK signaling pathway | O | O |
|  | HSA04910 | Insulin signaling pathway | O | X |
|  | HSA04510 | Focal adhesion | O | O |
|  | HSA04062 | Chemokine signaling pathway | O | X |
|  | HSA04370 | VEGF signaling pathway | X | X |

Table S3.
