# Supplementary material for: Pathway-Based Evaluation in Early Onset Colorectal Cancer Suggests Focal Adhesion and Immunosuppression along with Epithelial-Mesenchymal Transition
Source: PLoS One. 2012 Apr 9;7(4):e31685. doi: 10.1371/journal.pone.0031685 (PMC3322137; doi:10.1371/journal.pone.0031685)
Supplement: Table S5 — The expressions of TGF-βs and their receptors were summarized. Majority of the genes were up-regulated in the cancer except TGFBR1. (DOC) [file pone.0031685.s013.doc]

| Genes | Control^1^ | Cancer^1,2^ | Fold-change of cancer over control |
| --- | --- | --- | --- |
| TGF-β1 (TGFB1) | 7.841 | 9.229 | 2.617 |
| TGF-β2 (TGFB2) | 6.361 | 7.910 | 2.926 |
| TGF-β3 (TGFB3) | 7.836 | 8.690 | 1.808 |
| TGFBR1 (TGFB1 affinity) | 6.508 | 5.473 | 0.488 |
| TGFBR2 (TGFB1 affinity) | 11.662 | 12.451 | 1.728 |
| TGFBR3 (TGFB1, TGFB2 affinity) | 9.115 | 10.921 | 3.497 |

^1^It is the median of the log2-scaled expressions of the group.

^2^It is the normal-appearing mucosa in the CRC patients.

Table S5.
