# Supplementary material for: Pathway-Based Evaluation in Early Onset Colorectal Cancer Suggests Focal Adhesion and Immunosuppression along with Epithelial-Mesenchymal Transition
Source: PLoS One. 2012 Apr 9;7(4):e31685. doi: 10.1371/journal.pone.0031685 (PMC3322137; doi:10.1371/journal.pone.0031685)
Supplement: Appendix S1 — The additional analysis for GSEA comparison and independent dataset validation. (DOC) [file pone.0031685.s014.doc]

**CONTENTS (including its own tables and figures)**

Section 1. Comparison of our method and Gene Set Enrichment Analysis (GSEA) in the Hong dataset

Section 2. Comparison between the pathway substructure of the Hong et al. dataset and that of the other dataset

Section 1. **Comparison of our method and Gene Set Enrichment Analysis (GSEA) in the Hong dataset**

We performed GSEA for Hong et al.’s data and compared GSEA result with ours. However, we would like to point out that the main objectives of two methods are somewhat different, although there are some similarities between them (Table L-1). First, our method focuses on identification of subpathways (subsets of pathways) from KEGG pathway database, while GSEA identifies only pathways (gene sets) in the KEGG pathway database. Second, our method can account for regulations (e.g., activation, inhibition) between adjacent entries, while GSEA cannot.

Table L-1 Two important differences between the two methods

|  | Our method | GSEA |
| --- | --- | --- |
| Main objective | Identification of subpathways (subset of pathways) | Identification of pathways |
| Regulation (activation, inhibition) | Incorporated | Not incorporated |


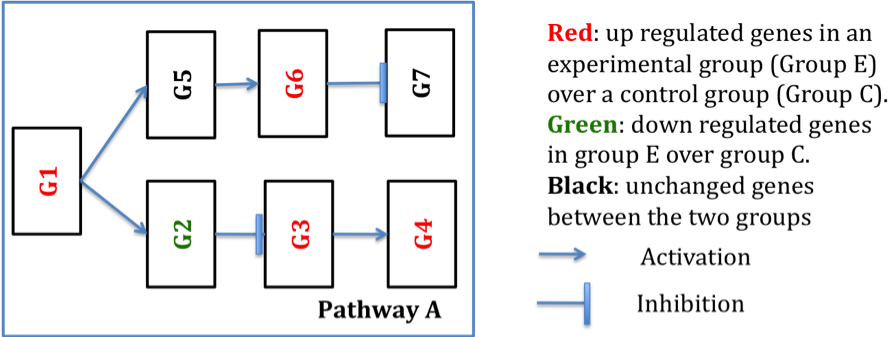


Figure L-1. A simple pathway example.

We illustrate the differences between the two methods by using a simple example shown in Figure L-1. Suppose the pathway with seven genes. Let the red color represent up-regulated genes in an experimental group (Group E) over a control group (Group C); the green color the down-regulated genes in group E over group C; the black color the unchanged genes between the two groups. The arrow represents activation and the blocked line inhibition. The GSEA result can show that Pathway A is activated in group E over group C. However, GSEA does not take regulation information (e.g., G1 and G2) into consideration. On the other hand, our method can provide the following information. (1) Pathway A is decomposed into two subpathways: G1->G5->G6-|G7 (subpathway A-1) and G1->G2-|G3->G4 (subpathway A-2); (2) the subset’s (G2-|G3->G4) expressions of subpathway A-2 agree with regulation flow (inhibition and activation from G2 to G4); (3) the expressions of subpathway A-1 do not agree with regulation flow.

Keeping these differences in mind, we compared the results of two methods in terms of the identified pathways. The KEGG pathways containing significant well-defined subpathways identified by our method were compared with those KEGG pathways obtained from the GSEA JAVA web start program (default options with 5,000 permutations). The significance level was 0.05 for the cutoff for the well-defined subpathways in our method and the same significance was applied to the GSEA method. Our method reported that 1,966 significant well-defined subpathways that corresponded to 78 KEGG pathways (Figure L-2). Also, the GSEA program reported two types of significant pathway lists: 10 activated pathways and 30 repressed pathways in the CRC patients (Figure L-3). The number of overlapped pathways between the two methods is six, which is not surprising after considering the differences between two methods. Nevertheless, the two methods identified six common cancer-associated pathways.

Figure L-2. The KEGG pathways containing 1,966 significant well-defined subpathways (p-value < 0.05) in our method. (# of subpaths: the number of the significant subpathways (p-value < 0.05) belonging to their corresponding KEGG pathway)


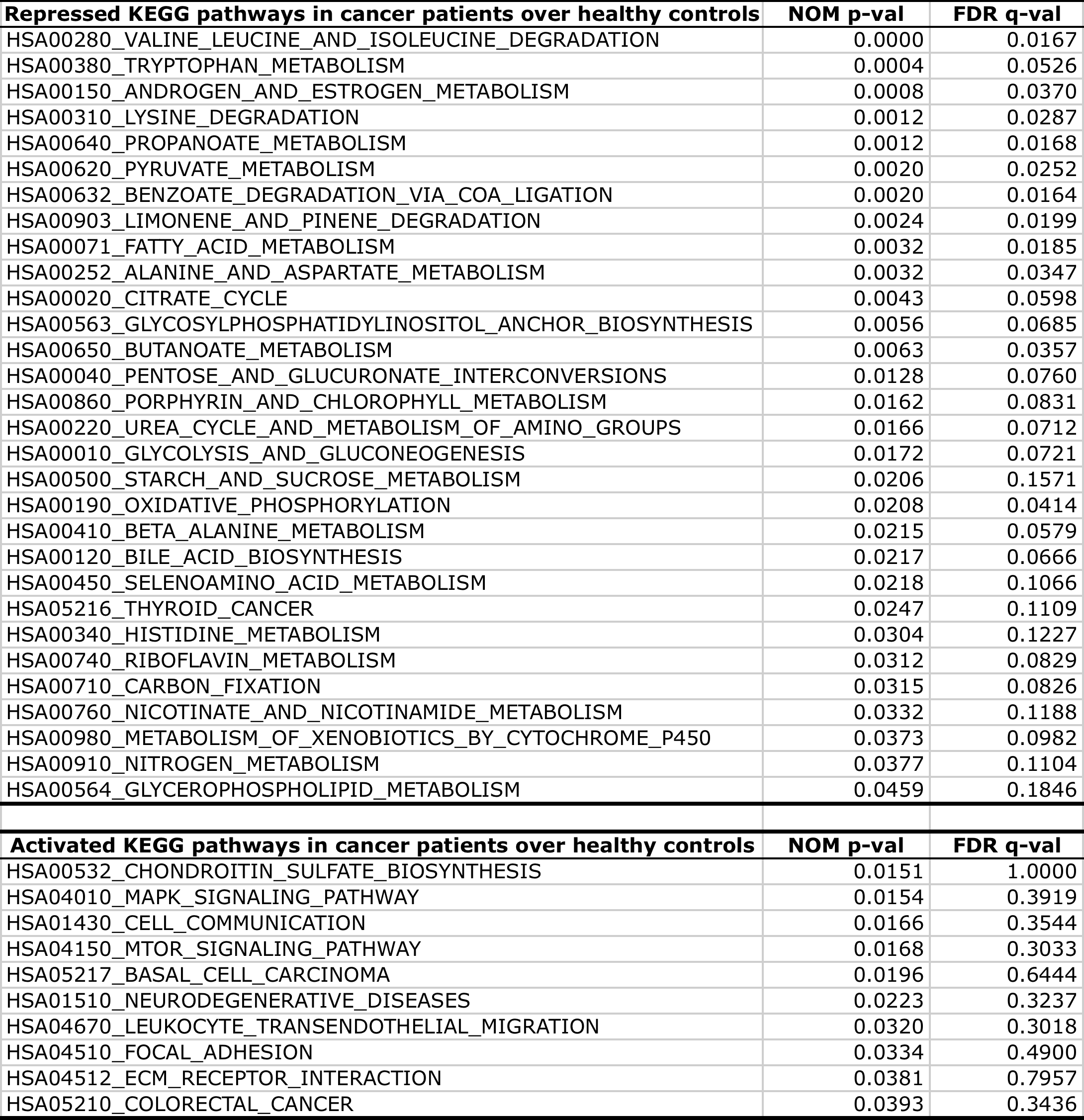


Figure L-3. The significant 40 KEGG pathways reported by GSEA in the Hong dataset. P-value (NOM p-val) less than 0.05 was used as the significance cutoff in the comparison analysis with GSEA.

In order to compare the 78 pathways identified by our method with the 40 pathways by GSEA, we used the cancer-related pathways reported by Vogelstein et al.[1] as a gold standard. We inspected which method provides more pathways consistent with the cancer-related pathways of Vogelstein et al. [1]. The cancer-related pathways [1] were manually mapped to their corresponding KEGG pathways because KEGG pathway identifiers corresponding to the cancer-related pathways were not mentioned explicitly in [1]. We inspected the overlapped pathways between the Vogelstein cancer-related KEGG pathways and those identified by the two methods. Table S3 shows that our method provides more consistent results with the Vogelstein cancer-related pathway than the GSEA method.

**Section 2. Comparison between the pathway substructure of the Hong et al. dataset and that of the other dataset**

| **Group** | **GSE4187 sample** |
| --- | --- |
| Colon normal (8 samples) | GSM95473 |
|  | GSM95474 |
|  | GSM95475 |
|  | GSM95476 |
|  | GSM95477 |
|  | GSM95478 |
|  | GSM95479 |
|  | GSM95480 |
| Colorectal carcinomas (15 samples) | GSM95496 |
|  | GSM95497 |
|  | GSM95498 |
|  | GSM95499 |
|  | GSM95500 |
|  | GSM95501 |
|  | GSM95502 |
|  | GSM95503 |
|  | GSM95504 |
|  | GSM95505 |
|  | GSM95506 |
|  | GSM95507 |
|  | GSM95508 |
|  | GSM95509 |
|  | GSM95510 |

Table L-2. GSE4187 sample description

We have searched additional colorectal dataset from GEO. Although there are several datasets for the colorectal cancer, no datasets are available relating to early onset colorectal cancer comparing early onset colorectal cancer patients with healthy controls, as Hong et al.’s dataset. Alternatively we were able to find a data set GSE4183 [2] comparing various colorectal diseases (colorectal carcinoma, colorectal adenoma, inflammatory bowel diseases) with normal control in a more general setting (www.ncbi.nlm.nih.gov/geo/query/acc.cgi?acc=GSE4183). From GSE4183 dataset, we obtained normal controls (8 healthy controls) and colorectal carcinomas (15 patients) (Table L-2). This new dataset (say GSE4183 if unambiguous) was analyzed by our method. 3,669 candidate well-defined subpathways (identified from ~130 million subpathways) were selected and their significances were determined based on 100,000 sample permutations.

In order to compare the pathway substructure of the Hong dataset with that of GSE4183, we focused on significantly identified subpathways from both dataset (“selection step” in Figure L-4 A), and inspected the intersection set of the two significantly identified subpathways (one for the Hong dataset, the other for GSE4183). In the comparison analysis, we set p-value 0.05 as the significance cutoff for both datasets. Since the well-defined subpathways with a small number of elements may provide too big intersection set, a more conservative selection step was applied for obtaining the intersection set (selection step in Figure L-4 A). The comparison showed that 250 well-defined subpathways were overlapped between the two datasets (Figure L-4 A). In order to determine how well these two results coincide with each other, we performed the Fisher’s exact test based on the randomization model. The p-value from the hyper-geometric distribution was less than 2.2e-16 (Figure L-4 B) implying that the two results coincided with each other well. Thus, we can conclude our finding on Hong’s pathway substructure is well supported by the finding from other independent dataset.


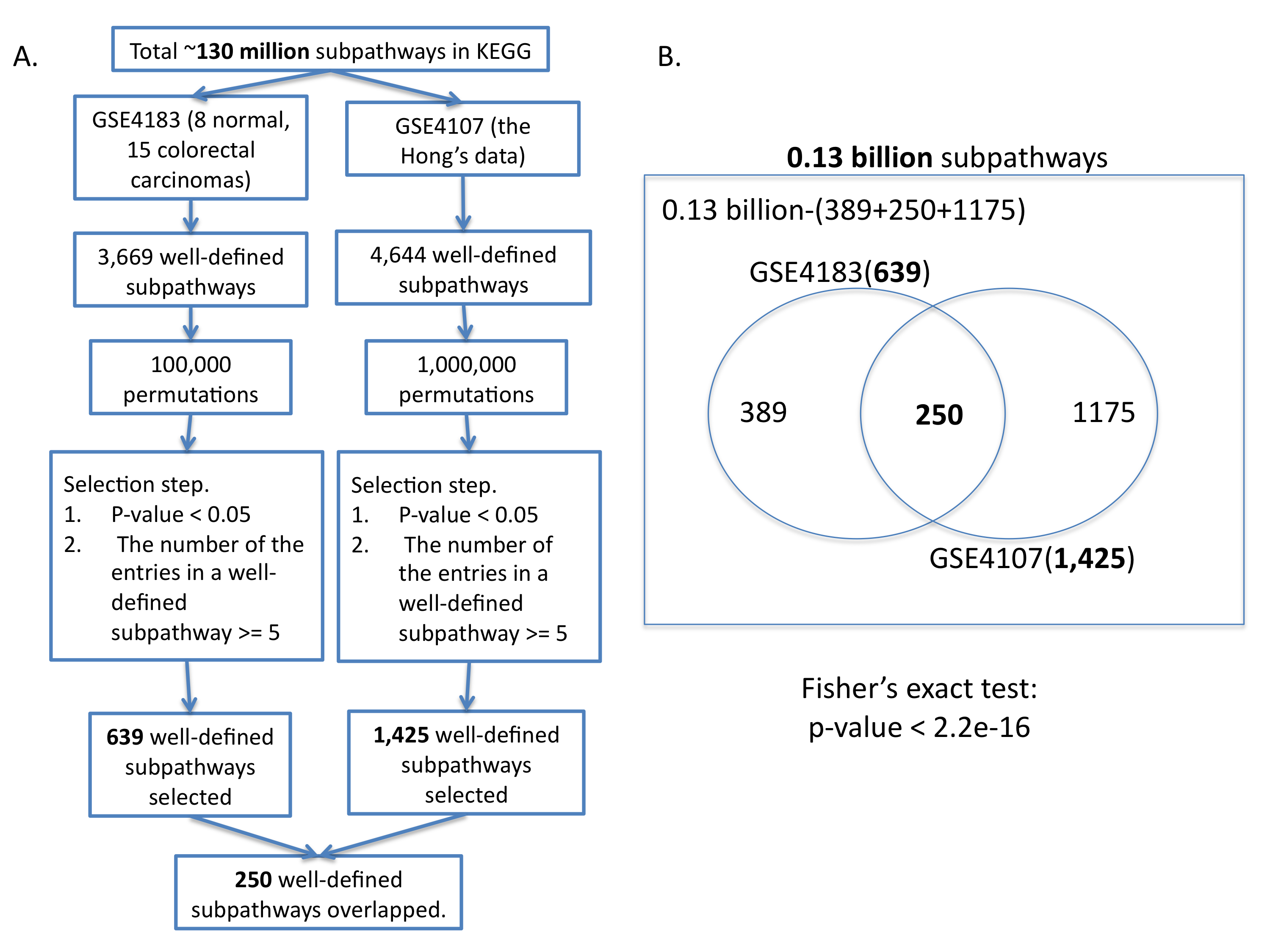


Figure L-4. Pathway substructure comparison between the Hong dataset and an independent dataset (GSE4183). A. The comparison procedure of the Hong dataset and GSE4183. B. The schematic diagram for measuring agreement of the pathway substructures between the two datasets. The bold number corresponds to that of A.

**References**

1. Vogelstein B, Kinzler KW (2004) Cancer genes and the pathways they control. Nat Med 10: 789-799.

2. Gyorffy B, Molnar B, Lage H, Szallasi Z, Eklund AC (2009) Evaluation of microarray preprocessing algorithms based on concordance with RT-PCR in clinical samples. PLoS One 4: e5645.
